# Complete Genome Analysis of *Thermus parvatiensis* and Comparative Genomics of *Thermus* spp. Provide Insights into Genetic Variability and Evolution of Natural Competence as Strategic Survival Attributes

**DOI:** 10.3389/fmicb.2017.01410

**Published:** 2017-07-27

**Authors:** Charu Tripathi, Harshita Mishra, Himani Khurana, Vatsala Dwivedi, Komal Kamra, Ram K. Negi, Rup Lal

**Affiliations:** ^1^Department of Zoology, University of Delhi New Delhi, India; ^2^Ciliate Biology Laboratory, Sri Guru Tegh Bahadar Khalsa College, University of Delhi New Delhi, India

**Keywords:** thermophiles, *Thermus parvatiensis*, CRISPR, *Pilus* genes, natural transformation, phage resistance

## Abstract

Thermophilic environments represent an interesting niche. Among thermophiles, the genus *Thermus* is among the most studied genera. In this study, we have sequenced the genome of *Thermus parvatiensis* strain RL, a thermophile isolated from Himalayan hot water springs (temperature >96°C) using PacBio RSII SMRT technique. The small genome (2.01 Mbp) comprises a chromosome (1.87 Mbp) and a plasmid (143 Kbp), designated in this study as pTP143. Annotation revealed a high number of repair genes, a squeezed genome but containing highly plastic plasmid with transposases, integrases, mobile elements and hypothetical proteins (44%). We performed a comparative genomic study of the group *Thermus* with an aim of analysing the phylogenetic relatedness as well as niche specific attributes prevalent among the group. We compared the reference genome RL with 16 *Thermus* genomes to assess their phylogenetic relationships based on 16S rRNA gene sequences, average nucleotide identity (ANI), conserved marker genes (31 and 400), pan genome and tetranucleotide frequency. The core genome of the analyzed genomes contained 1,177 core genes and many singleton genes were detected in individual genomes, reflecting a conserved core but adaptive pan repertoire. We demonstrated the presence of metagenomic islands (chromosome:5, plasmid:5) by recruiting raw metagenomic data (from the same niche) against the genomic replicons of *T. parvatiensis*. We also dissected the CRISPR loci wide all genomes and found widespread presence of this system across *Thermus* genomes. Additionally, we performed a comparative analysis of competence loci wide *Thermus* genomes and found evidence for recent horizontal acquisition of the locus and continued dispersal among members reflecting that natural competence is a beneficial survival trait among *Thermus* members and its acquisition depicts unending evolution in order to accomplish optimal fitness.

## Introduction

The genus *Thermus* belongs to the vast group of extreme thermophiles that have held biochemical and industrial attention. The discovery of *Thermus aquaticus* in 1969 (Brock and Freeze, 1969), and subsequently the multi-billion-dollar industry of *Taq* DNA polymerase has revolutionized the field of extremophile research. Not only do the extremophiles provide understanding of life at extreme habitats, but they also serve as model organisms to study protein structure and functions. Members of this genus have been isolated from hot water springs all over the world (Chung et al., 2000; Ming et al., 2014). The proteins encoded by *Thermus* spp. have high stability and have been used in various industries, DNA polymerases (Carballeira et al., 1990; Engelke et al., 1990; Rao and Saunders, 1992) being chief among them; along with xylanases (Blank et al., 2014), amylases (Shaw et al., 1995), lipases (Kretza et al., 2012) and many other enzymes. The genus is well known for bioremediation of heavy metals thus lowering the toxicity at heavy metal contaminated sites. *T. scotoductus* for instance, has been shown to reduce Cr(VI) aerobically (Opperman and van Heerden, 2006).

The phylogenetic relationships amongst the members of the genus have been as dynamic as the genetic constitution of its members (Kumwenda et al., 2014). Members of the genus *Meiothermus*, which were earlier classified to the genus *Thermus*, were later moved to constitute a new genus to accommodate these moderately thermophilic and strictly aerobic group (Nobre et al., 1996). The members of the genus *Thermus* are a part of the phylum Deinococcus-Thermus, along with *Deinococcus, Deinobacter, Deinobacterium, Truepera, Marinithermus, Meiothermus, Oceanithermus, Rhabdothermus*, and *Vulcanithermus*. Members of the genus *Thermus* form yellow to orange-yellow colonies and generally have small genome sizes of less than 2.5 Mb with extrachromosomal elements of common occurrence (Henne et al., 2004; Bruggemann and Chen, 2006). Previous genome analyses of the group have revealed highly plastic nature of the genome of *Thermus* species with plasmids and megaplasmids being the center for such plasticity (Bruggemann and Chen, 2006). Genome wide rearrangements have been instrumental in shaping the genomes of these thermophiles (Kumwenda et al., 2014). Elements that are considered to belong to the mobilome, i.e., insertion sequence (IS) elements, transposons and prophages, occur widely in the genomes of *Thermus* species (Kumwenda et al., 2014). Along with this, thermophilic organisms are known to thrive under viral selection pressure. Selective forces continually acting at extreme environments bring about a strong evolutionary streamlining of these genomes (Sabath et al., 2013).

Among the drivers of evolution; genetic recombination, rearrangement, horizontal gene transfer, conjugation, transformation and mutations are key players for this genus (Kumwenda et al., 2014). One of the most likely explanations for this is the highly evolved competence system of *Thermus* which serves as an efficient arrangement for the uptake of alien genetic material from the environment (Lorenz and Wackernagel, 1994). In most organisms, competence is not a constitutive phenomenon, but tightly controlled by factors related to the cell cycle (induced/artificial competence). In contrast to artificial competence, some organisms (including *Thermus* species) are constitutively competent (Friedrich et al., 2002); reviewed by Averhoff (2009). The exact mechanism of uptake of free DNA from the environment varies from species to species. In case of *Thermus* species, the type IV pilus (T4P) system has been implicated in natural transformation, although the link between piliation and natural transformation seems unclear. Amongst all naturally competent species, *Thermus thermophilus* HB27 has the most efficient (40 kb/s per cell) natural competence system and a robust, non-selective competence machinery (Averhoff, 2009). The development of competence machinery in *Thermus* is of great evolutionary significance and explains the dynamism in *Thermus* genomes. We provide a brief introduction of the genes involved in imparting natural competence, followed by a genus-wide analysis of competence loci in *Thermus*.

The genus *Thermus* comprises 17 validly published species, the genome sequences of which are available in public databases. *T. parvatiensis* strain RL^T^ (Dwivedi et al., 2015) was isolated from a hot water spring located atop (altitude ~1,700 m) the Himalayan ranges at Manikaran, India. The hot spring water has high temperature (90–98°C) (Dwivedi et al., 2012) and circum-neutral pH. Low O_2_ potential (4.8 ± 0.2 cm^3^ STP/L), low dissolved CO_2_ (14.7 ± 0.1 cm^3^ STP/L) and high concentration of arsenic (140 ppb) (Sangwan et al., 2015) prevalent at the niche further provide strong selection pressures. *T. parvatiensis* forms yellow colonies on polypeptone yeast extract agar at 60-80°C and demonstrates protease activity (Dwivedi et al., 2015). Previously, strain RL was sequenced using Roche 454 GS (FLX Titanium) system and Sanger shotgun sequencing. The raw data generated was assembled into 17 contigs (Dwivedi et al., 2012). In order to fill the gaps and generate a complete genome record, we determined the entire genome sequence using single molecule real time (SMRT) sequencing method. Here, we present the complete genome of *T. parvatiensis* and perform a comparative genomic analysis of available *Thermus* genomes. Our study is designed to uncover the phylogenetic relatedness among members based on phylogenomic methods, the core-pan genome structure as well as conserved genome features with the help of metagenomic recruitments. Further, we have analyzed genus specific evolutionary dynamics which are facilitated by the highly efficient natural competence system of this genus and a possible link with predominance of viral signatures found in these genomes.

## Materials and methods

### Genome sequencing, assembly, and annotation of *T. parvatiensis* replicons

SMRT genome sequencing was performed using PacBio RSII system at McGill University and Genome Quebec Innovation Centre, Canada. Genomic DNA was extracted using CTAB method (Doyle and Doyle, 1990) followed by quality assessment on gel and quantification by ND1000 Nanodrop spectrophotometer. Sheared large insert library preparation was followed by generation of raw reads with an average read length of 9,878 nt. A total of 2,488 GB raw data was generated with 224,211 reads encompassing 857,926,800 bases with an average sequencing depth of 428 × (Supplementary Table 1). *De novo* assembly was performed at the Genome Quebec, Canada using the HGAP assembler (Chin et al., 2013) (coverage cut-off 30 ×). Assembly validation was performed by aligning raw reads onto finished contigs using the Burrows-Wheeler Aligner version 0.7.9a (Li and Durbin, 2009). Visual inspection of the assembly was performed using Tablet version 1.14.04.10 (Milne et al., 2010). Ends of contigs were searched for overlaps using the formatdb and BLAST functions of Ugene (Okonechnikov et al., 2012). Circularized replicons were uploaded on RAST server (Aziz et al., 2008) for general genome annotations. RNAmmer version 1.2 (Lagesan et al., 2007) was used to detect rRNA operons. Phages were scanned using online tools PHAST (Zhou et al., 2011) and PHASTER (Arndt et al., 2016). For detailed annotations of the phage regions, analysis was extended to include probable phages and associated regions using Phage Search Tool against viral and prophage databases (http://www.phantome.org/Downloads/). Further, PHAST tool was also used to decipher the completeness of the phage genome followed by annotation using BLASTx against the ORFs predicted from prophage databases. Aragorn (Laslett and Canback, 2004) online tRNA database was used to detect tRNAs in the genome. The WebMGA (Wu et al., 2011) server was used for general COG category assignment. An approach integrating the Z-curve analysis, dnaA box location and genes surrounding the OriC was used to identify the origin of replication on the chromosome using the Ori-Finder server (Gao and Zhang, 2008). *T. parvatiensis* genome was searched against DNA box database to locate the origin of replication and for this, DNA box repeat sequence (TGTGGATAA) of *T. thermophilus* (closest relative of *T. parvatiensis*) was used as reference to guide the BLAST search.

### Phylogenomic assessments

For comparative analyses, sequenced genomes of the genus *Thermus* (17 genomes) were downloaded from the NCBI GenBank database. The genomes included in this study are, *T. thermophilus* HB27 (Henne et al., 2004), *T. thermophilus* HB8, *T. parvatiensis* RL (Dwivedi et al., 2012, 2015), *T. scotoductus* SA-01 (Gounder et al., 2011), *T. oshimai* JL-2 (Murugapiran et al., 2013), *Thermus* species CCB_US3_UF1 (Teh et al., 2012), *T. aquaticus* Y51MC23, *T. antranikianii* HN3-7, *T. filiformis* Wai33 A1, *T. thermophilus* JL-18 (Murugapiran et al., 2013), *T. thermophilus* SG0.5JP17-16, *T. islandicus* PRI3838, *T. caliditerrae* YIM 77777, *T. igniterrae* RF-4, *T. amyloliquefaciens* YIM 77409 (Yu et al., 2015; Zhou et al., 2016), *T. tengchongensis* YIM 77401 (Mefferd et al., 2016), and *T. brockianus* GE-1. Accession numbers of genomes included in this study and other general genome features are included in Table 1. Among the genomes selected for comparisons, 10 were complete genomes and seven were draft genomes. All complete genomes selected were found to harbor 1–4 plasmid(s) which were downloaded as separate sequences.

**Table 1:** General genome features of organisms belonging to the genus *Thermus*.

	**Strain**	**NCBI Accession No**.	**Source of Isolation**	**Genome Size**	**Plasmid (s)**	**G+C (%)**	**Predicted CDS**	**tRNA**	**rRNA operons**
*T. parvatiensis*	RL^*^	CP014141	Hot spring, India	2,016,098	pTP143 (143,277)	68.5	2,383	54	2
*T. thermophilus*	HB27^*^	AE017221	Hot spring, Japan	2,127,482	pTT27 (232,605)	**69.4**	2,244	47	6
*T. thermophilus*	HB8^*^	AP008226	Hot spring, Japan	2,197,207	pTT27 (256,992), pTT8 (9,322), pVV8 (81,151)	**69.4**	2,268	48	6
*T. thermophilus*	JL-18^*^	CP003252	Great Boiling Spring, USA	2,311,212	pTTJL1801 (265,886), pTTJL1802 (142,731)	69.0	2,424	52	6
*T. thermophilus*	SG0.5JP17-16^*^	CP002777	Hot Spring	2,303,227	pTHTHE1601 (**440,026**)	68.6	2,405	53	6
*T. scotoductus*	SA-01^*^	CP001962	Fissure water, South Africa	2,355,186	pTSC8 (8,383)	64.9	2,514	47	6
*T. oshimai*	JL-2^*^	CP003249	Great Boiling Spring, USA	2,401,329	pTHEOS01 (271,713), pTHEOS02 (57,223)	68.6	2,521	59	6
*T*. sp.	CCB_US3_UF1^*^	CP003126	Hot spring, Malaysia	2,263,488	pTCCB09 (19,716)	68.6	2,228	48	6
*T. aquaticus*	Y51MC23^*^	CP010822	Hot spring, USA	2,338,641	pTA14 (14,448), pTA16 (16,597), pTA69 (69,906), pTA78 (78,727)	68.0	2,436	55	3
*T. brockianus*	GE-1^*^	CP016312	Kamchatka, Russia	2,388,273	pTB1 (342,792), pTB2 (10,299)	66.9	2,789	47	2
*T. antranikianii*	DSM 12462 (HN3-7)	AUIW01000000	Hot spring, Iceland	2,163,625	ND	64.8	2,321	47	4
*T. filiformis*	ATCC 43280 (Wai33 A1)	JPSL02000000	Hot spring, New Zealand	2,386,081	ND	69.0	2,338	47	6
*T. islandicus*	DSM 21543 (PRI 3838)	ATXJ01000000	Hot spring, Iceland	2,263,010	ND	68.4	2,470	47	6
*T. igniterrae*	ATCC 700962 (RF-4)	AQWU01000000	Hot spring, Iceland	2,225,983	ND	68.8	2,379	43	6
*T. caliditerrae*	YIM 77777	JQNC01000000	Hot spring, China	2,218,114	ND	67.2	2,327	50	3
*T. amyloliquefaciens*	YIM 77409	JQMV00000000	Hot spring, China	2,160,855	ND	67.4	2,313	48	6
*T. tengchongensis*	YIM 77401	JQLK01000000	Geothermally heated soil, China	**2,562,314**	ND	66.4	2,750	47	2

*Smallest genome/plasmid sequences and GC content are underlined whereas the largest are in bold*.

* *Complete genome; ND-Not determined*.

Phylogenetic analysis based on traditional 16S rRNA gene sequences was performed, for which 16S rRNA genes were fetched from the respective genomes using RNAmmer version 1.2 server (Lagesan et al., 2007). Multiple sequence alignment was performed using Muscle (Edgar, 2004). Unaligned sequences were trimmed from the edges. Phylogenetic tree was constructed using Maximum-likelihood (ML) algorithm (Felsenstein, 1993) employed in Mega version 6 (Tamura et al., 2013). Although 16S rRNA gene is a well-established marker for tracing phylogeny, dependence on just one gene may lead to biased phylogenetic projections. Hence, evolutionary relationships were reconstructed using multiple conserved marker genes extracted from the genomes. For this, 31 conserved bacterial single copy genes were extracted from each genome using AmphoraNet server (Kerepesi et al., 2014). Individual marker gene sequences for individual genomes were concatenated. Alignment was performed using Muscle (Edgar, 2004). Further, 400 conserved bacterial marker genes were retrieved from each genome using PhyloPhlAn (Segata et al., 2013). For the above three sequence based analyses, ML trees were rendered with 1,000 bootstrap revaluations. To trace phylogeny using whole genome data, well established phylogenomic approaches were employed. Average nucleotide identity (ANI) values were calculated using the BLASTALL algorithm (ANIb) of JSpecies v 1.2.1 (Richter and Rosello-Mora, 2009). A two-way matrix containing pairwise ANI scores was used to perform hierarchical clustering using Pearson correlation (average linkage). Similar dendrogram was generated using a two-way matrix of tetranucleotide frequencies calculated using regression analysis by JSpecies v 1.2.1. To evaluate phylogeny on the basis of variable component of the genome, pan genome phylogeny was reconstructed by hierarchical clustering using information from a binary gene presence-absence (1/0) matrix generated by BPGA (Chaudhari et al., 2016). Gene presence-absence matrix constituted the information about presence or absence of the total gene complement (pan genome) for all *Thermus* species. In order to resolve the precariousness of sub-species level relationships, pairwise digital DNA-DNA hybridization (dDDH) values were calculated using the genome to genome distance calculator (ggdc.dsmz.de) (Auch et al., 2010).

### Analysis of genome flexibility

Genome sequences were uploaded on RAST server (Aziz et al., 2008) and coding sequences were extracted from RAST predictions. Coding sequences (amino acids) were compared using formatdb and BLASTALL programs available in the package BLAST version 2.2.26 (Altschul et al., 1990). Genomic islands were predicted using IslandViewer 3 (Dhillon et al., 2015). Dot-plots and synteny maps were constructed to uncover the extent of rearrangements (duplications, deletions, insertions) occurring as a function of genome distance. Dot-plots were generated using BLASTN (Wheeler and Bhagwat, 2007) with *T. parvatiensis* as the reference. Synteny maps were constructed by identifying conserved locally collinear blocks (LCBs) among genomes, followed by whole genome alignments using progressiveMauve version 20150226 (Darling et al., 2010) at three spaced seed patterns and a high seed weight (seed weight = 15) for sensitive alignment of closely related genomes. Horizontally acquired regions on the megaplasmid pTP143 were detected by BLAST based comparison with all sequenced *Thermus* plasmids. These regions were confirmed using Alien Hunter (Vernikos and Parkhill, 2006) at default thresholds. Further, a mapping of syntenic regions on all *Thermus* plasmids was performed using progressiveMauve (Darling et al., 2010) for visual demonstration.

Core and pan genome analysis was performed using BPGA algorithm (Chaudhari et al., 2016). Usearch (Edgar, 2010), which is the default clustering algorithm of BPGA, was employed for orthologous gene identification and clustering. Core genome plot was rendered by plotting the total number of shared genes with each subsequent addition of a genome against the number of genomes. Pan genome plot was rendered by plotting the total number of distinct gene families identified with the addition of each genome vs. number of genomes. To avoid biasedness, median values of 20 random permutations were used for rendering these plots. Representative (seed) sequences of both core and pan genome were used for function based analysis.

Metagenomic sequence data recruitment was performed in order to gain insights into strain specific flexible repertoire harbored by *T. parvatiensis*, and to investigate whether this flexibility is strain specific or extended to other members of the genus as well. For this purpose, water samples were collected from the hot water spring at Manikaran, India, at two locations, namely MNW1 and MNW2 (intra-site distance: 100 m; 32°01′34.8″N, 077°20′50.3″E). DNA extraction from the water samples (10 L each, filtered through 0.45 μ filters) was carried out by PowerMax (R) Water DNA isolation kit (MoBio Laboratories Inc., Carlsbad, CA, USA) as per manufacturer's instructions. Metagenomic data was generated using Illumina GAII technology with an insert size of 170 bp (DDBJ/EMBL/GenBank accession number PRJEB19501). In order to delineate metagenomic islands (hereafter referred to as MGIs), metagenomic raw reads from hot spring water (MNW1 and MNW2) were recruited onto the chromosome and plasmid of *T. parvatiensis* using nucmer available with MUMmer (Kurtz et al., 2004) package. Coverage plots were generated using mummerplot available in MUMmer package. Regions with no or little mapping after metagenomic reads tilling at coverage cut-off of 80% were identified as MGIs (Steffen et al., 2012). Mapping coverage was determined using coords file generated by nucmer (identity cut-off: 80%). BLASTp algorithm was implemented to identify the presence of these regions on other *Thermus* genomes.

### Detection of genus specific survival strategies

#### CRISPR analysis

CRISPR arrays were extracted from genomes using CRISPRFinder (Grissa et al., 2007) online server which performs BLAST against dbCRISPR (CRISPR database; last updated on 2017-01-02). CRISPRFinder further classifies the identified CRISPR arrays as true or false based on whether or not they are associated with CRISPR associated genes (*Cas*) respectively. *Cas* genes were annotated using CRISPRone (Zhang and Ye, 2017). CRISPRs lacking *Cas* genes in the vicinity were designated as false/questionable CRISPRs. Only true CRISPRs were selected for analyses. CRISPR arrays have two components: repeats and spacers; both of which were analyzed to study the evolution and probable viral diversity respectively. Classification and clustering of CRISPR repeats and repeat-based *Cas* gene predictions were undertaken using CRISPRmap, a comprehensive cluster analysis method (based on Markov clustering) which clusters conserved sequence families and potential structure motifs (Lange et al., 2013). Repeats were classified based on 40 conserved sequence families and 33 probable structural motifs. Further, 24 families and 18 structural motifs were considered for the construction of repeat cluster maps. For prediction of potential viruses most frequently associated with *Thermus* genomes, spacer sequences from all genomes were extracted and BLAST against viral GenBank database of NCBI (Deng et al., 2007) with a threshold e-value of 1. For better stringency, among all matches, only those having 100% identity of more than 20 nucleotides were considered as valid hits.

#### Comparison of competence imparting genes

Genes involved in imparting competence (16 genes) to *T. thermophilus* HB27 were used as reference for extracting competence associated genes from individual genomes using BLAST. *PilA1-A4* genes were aligned using Hirschberg (KAlign) algorithm (Lassmann and Sonnhammer, 2005). Visual alignment consensus was built at 70% threshold. Relationships among *PilA1-A4* genes were inferred by PhyML (Guindon et al., 2009) maximum-likelihood method using HKY85 substitution model (Hasegawa et al., 1985). Median size estimations were made using boxplot function in R (https://cran.r-project.org/mirrors.html).

## Results and discussion

### Genome sequencing, assembly, and annotation of *T. parvatiensis* replicons

#### Genome assembly, finishing, and annotation

**Figure 1:**
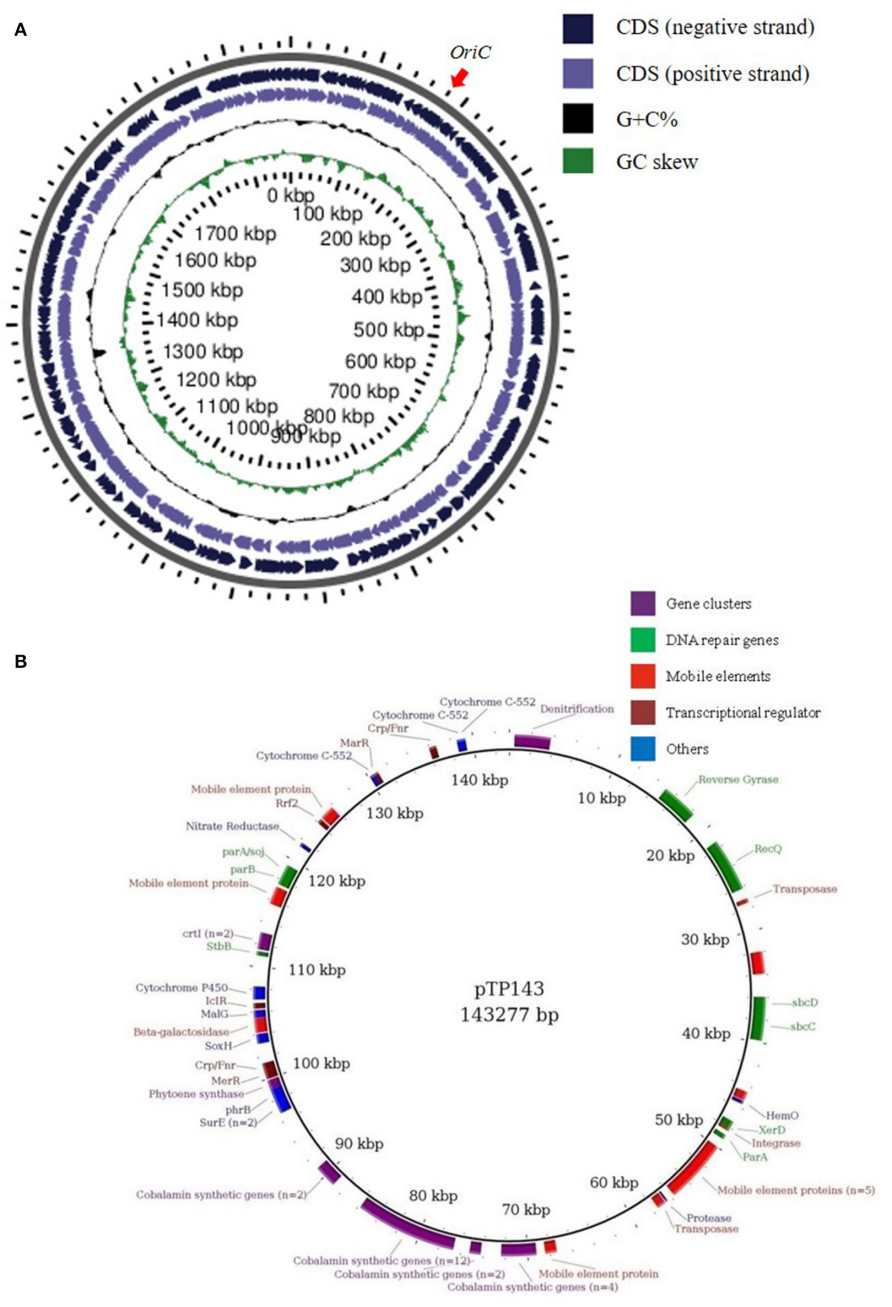
Replicon maps of *Thermus parvatiensis* strain RL **(A)** ORFs on the chromosome have been mapped on both strands, the origin of replication is marked with a red arrow. From outside to inside: genes on the negative strand, genes on the positive strand, GC percentage and GC skew. **(B)** Detailed map of *T. parvatiensis* megaplasmid pTP143 marked with prominent categories of genes in different colors. Genes representing DNA repair genes (green), mobile element genes (red), transcriptional regulators (magenta) and gene clusters (purple; denitrification gene cluster, cobalamin biosynthetic gene cluster, carotenoid synthesis gene cluster) have been specifically highlighted.

The genome of *T. parvatiensis* strain RL was initially assembled into three contigs (totally 2,066,435 bp, 1,886,121 bp, 159 Kbp and 21 Kbp) with G+C content of 68.5%. The 21 Kbp contig, mapped onto the chromosome (BLASTn and mapping with previously generated assembly; Dwivedi et al., 2012). The entire 21 Kbp region, seems to represent an integrated plasmid or a large genomic island incorporated into the genome, based on the annotation of mostly hypothetical genes and transposable elements among genes identified. This was supplemented by differences in mean G+C% of this region (67.3%) as compared to the rest of the genome (68.5%). In an attempt to circularize the largest contig, its ends were aligned against each other and an overlapping region of 13,300 nt was removed. Similarly, in order to circularize the 159 Kbp contig, an overlapping region of 15,853 bp region was removed from the ends of the contig. Finally, two replicons were reconstructed: a chromosome (1,872,821 bp) and a megaplasmid (143,277 bp) (Figure 1) (total size: 2,016,098 bp) (Table 2). The chromosomal origin of replication was located at 158,478–158,778. A total of 12 DNA boxes with consensus sequence of TGTGGATAA were identified spanning the 301 nt *OriC* region. The total number of predicted coding sequences were 2,383. The genome was found to harbor two rRNA operons and 54 tRNAs and tmRNAs. COG functional category assignment placed a large number of genes to amino acid transport and metabolism (11.04%), general function prediction (12.95%), energy production and conservation (7.54%) and translation, ribosomal structure and biogenesis (7.40%). A number of genes were classified into the unknown function category (7.78%). A large proportion of the genome is strictly attributed to genes needed by the organism for essential cellular processes. *T. parvatiensis* thrives at a high arsenic concentration (140 ppb). We investigated the presence of arsenic resistance mechanisms in this thermophilic organism. Arsenate reductase gene *arsC* (1 copy), arsenic efflux pump protein *arsB* (2 copies) and *arsR* transcriptional regulator (4 copies) were identified. The above genes belong to the ars (arsenic resistance) operon responsible for the efflux of As(III) out of the cells (Yang and Rosen, 2016). Genes for the oxidation of arsenic (aox operon) were not identified. The mechanism of arsenic detoxification in *T. parvatiensis* thus involves extrusion of arsenic out of the cells (rather than oxidation).

**Table 2:** Genomic features of the chromosome and plasmid of *T. parvatiensis* strain RL.

	**Chromosome**	**Plasmid**
Accession number	CP014141	CP014142
Size (bp)	1,872,821	143,277
G+C content (%)	68.5	68.4
CDS	2326	57
Coding density (%)	94.12	87.65
tRNAs	54	0
rRNA operons	2	0

*T. parvatiensis* strain RL uniquely harbored three integrated phages in its genome (Supplementary Figure 1)—two on the chromosome and one on the plasmid. This was in contrast to all its close phylogenetic neighbors taken into account in this study namely, *T. thermophilus* HB8, *T. thermophilus* HB27, *T. thermophilus* JL18, and *T. thermophilus* SG0.5JP17-16 in which no phages could be identified. The first phage (20.3 Kbp) on the chromosome revealed the presence of phage structural proteins such as tail assembly protein and coat protein along with three heat shock proteins which can be directly implicated to the environment, i.e., hot spring water (surface water temperature >95°C). The phage region was associated with two hybrid histidine kinases, which are implicated in two-component regulatory system (Khorchid and Ikura, 2006). The second chromosomal phage (23.2 Kbp) was annotated and revealed a probable integron with attL and attR sites along with integrase encoding gene and flanking tRNA. The gene cassette of this integron had 23 hypothetical proteins and 16 viral proteins. These results also indicate that this integron might denote a super-integron with 37 ORFs captured. Phage regions have been known to play a role in horizontal gene transfer by specialized as well as generalized transduction (Touchon et al., 2017). Genes harbored on the integrated phage regions corresponding to two-component system and heat shock proteins reflect the dispersal of these genes might be an active phenomenon among the population.

#### Plasmid pTP143

Plasmids, including small plasmids, as well as large megaplasmids of up to 440 Kbp are known to be present in *Thermus* genomes (Table 1). The plasmids of *Thermus* are known to be the center of plasticity, harboring genes for mobile elements, transposons and a number of biosynthetic clusters (Henne et al., 2004; Bruggemann and Chen, 2006). The megaplasmid pTP143 of *T. parvatiensis*, contained 181 coding sequences. A large number of the genes harbored on the plasmid were, however, genes belonging to integrases, transposases, mobile elements and hypothetical protein coding genes (81 genes, constituting 44% of the total plasmid genes) (Figure 1) which denote the plastic nature of the plasmid. A low coding density (87.65%) was observed for pTP143, as compared to the chromosome (94.12%). Genetic analysis of *T. parvatiensis* megaplasmid pTP143 revealed a cobalamin biosynthetic cluster, a denitrification cluster and a carotenoid biosynthesis cluster (responsible for imparting the yellow pigment to the organism). A thermophilic lifestyle demands a robust DNA repair system. Genes required for thermophilic existence most suitably correspond to an elevated number of DNA repair genes (Bruggemann and Chen, 2006). Consequently, a *recQ* helicase, reverse gyrases, photolyase *phrB, sbcC*, and *sbcD* nucleases (implicated in deleting hairpin structures) were found on the megaplasmid. Genes related to stress response, *surE* (involved in nucleic acid pool maintenance; Proudfoot et al., 2004) and cytochrome P450 (Kelly and Kelly, 2013) were also identified. A number of transcriptional factors known to modulate stress conditions were found on the plasmid. These included a transcriptional regulator *IcIR* involved in regulation of responses to quorum sensing and toxic stress (Molina-Henares et al., 2005). A transcriptional regulator *Crp/Fnr* known to be responsive to environmental changes (Körner et al., 2003; Zhou et al., 2012), such as oxidative stress, carbon dioxide concentrations and heavy metal impositions was annotated. *Crp/Fnr* regulators act by regulating the expression of genes involved in alleviating the respective stress conditions. *MerR*, a heavy metal modulating transcriptional regulator (Brown et al., 2003), which activates promotors of genes in response to heavy metal influx was annotated. *T. parvatiensis* harbors a plastic plasmid with mobile and hypothetical gene components. Constituting genes for DNA repair, stress response and transcriptional regulators, we believe that the megaplasmid has an indispensable role to play for the thermophilic survival of *T. parvatiensis*. Not only essential for survival, the megaplasmid demonstrates a potentially crucial role in communicating and modulating temperature stress via an appropriate response carried out by the transcriptional regulators it harbors.

### Phylogenomic assessments

**Figure 2:**
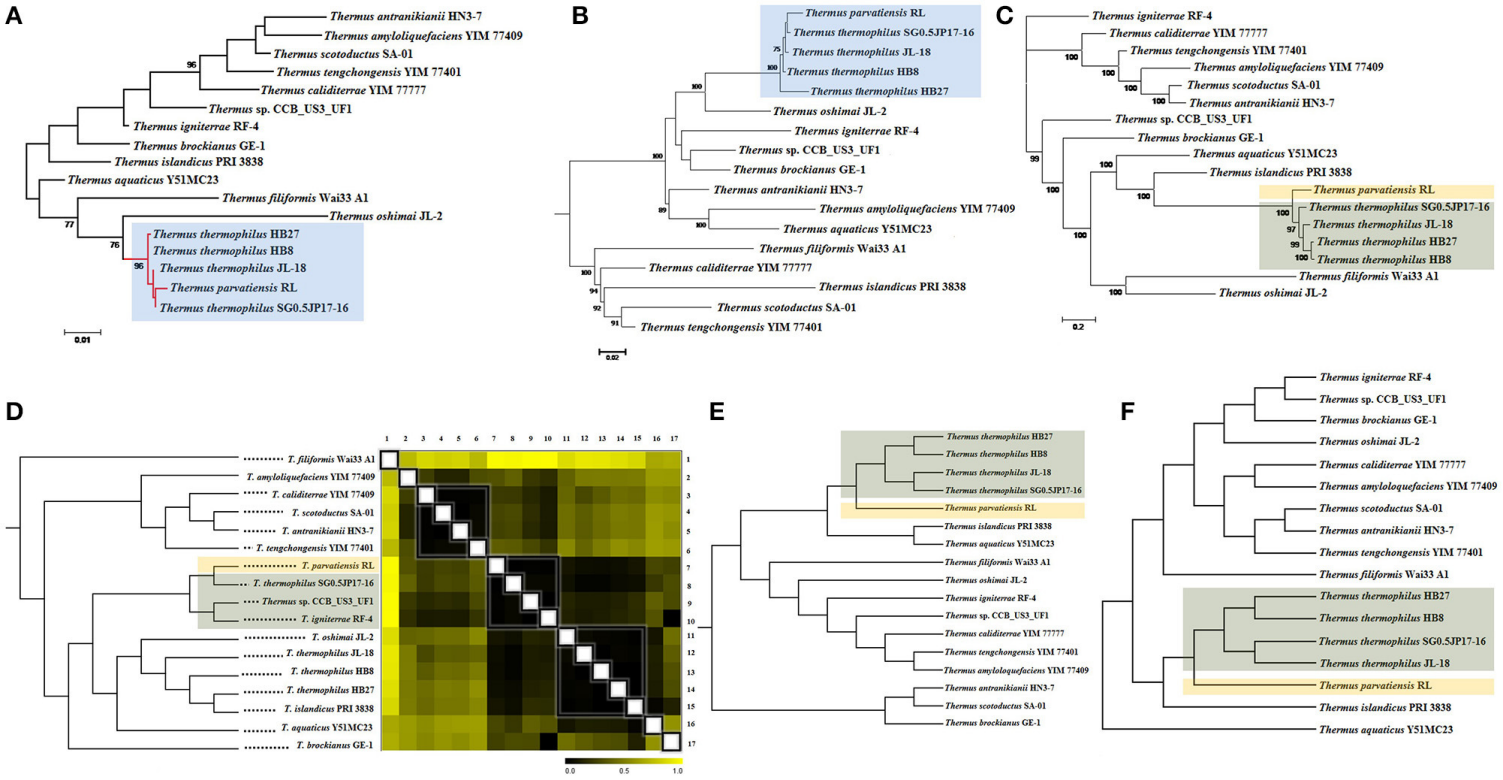
Inference of evolutionary relationships among *Thermus* spp. based on phylogenetic and phylogenomic methods. Phylogenetic analysis based on **(A)** 16S rRNA gene sequences; **(B)** 31 single copy genes; and **(C)** 400 conserved bacterial marker genes, of the species under study using maximum likelihood method. Bars represent the number of substitutions per nucleotide position. Percentage bootstrap values (≥70%) are shown next to the nodes. Phylogenomic dendrograms showing hierarchical clustering of species under study constructed using **(D)** whole genome distance matrix based on ANI scores; **(E)** tetranucleotide frequencies; and **(F)** pan genes presence-absence matrix. Gradation of colors from black to yellow in **(D)** depicts increasing genome distance on the basis two-way ANI matrix. Black denotes minimum distance and yellow denotes maximum distance. Organisms have been grouped together into clades on the basis of minimum distance (black). Blue shade (in **A,B**) depicts clustering of *T. parvatiensis* within *T. thermophilus* group. Light brown shade (in **C–F)** depicts the position of *T. parvatiensis* separately from other closely clustered *Thermus* members (shaded gray in **C–F)**.

The novel phylogeny of strain RL has already been discussed based on multi locus gene analysis (Dwivedi et al., 2015). We describe here, the microbial phylogeny within the genus *Thermus* using the traditional 16S rRNA gene, 31 bacterial single copy genes and 400 conserved bacterial marker genes. To strengthen the analyses, we have used whole genome patterns established by ANI scores, tetra-nucleotide scores, pan genome and dDDH values. The phylogenetic tree based on 16S rRNA gene sequences placed strain RL along with members of the *T. thermophilus* clade into a single monophyletic clade, closely bifurcating with SG0.5JP17-16 (Figure 2), and residing with strains of *T. thermophilus*, i.e., HB27, HB8, JL-18, and SG0.5JP17-16. This was expected from over 99% identity of 16S rRNA gene sequence of *T. parvatiensis* with members of *T. thermophilus*. A single gene, however, is not able to provide the required phylogenetic resolution, hence, phylogenetic relationships were further investigated on the basis of conserved marker genes. For this, 31 bacterial single copy genes and 400 bacterial conserved marker genes were used. The phylogenetic tree constructed using 31 essential single copy genes placed *T. parvatiensis* in the same clade as *T. thermophilus* (Figure 2). However, the phylogenetic tree constructed using 400 conserved bacterial marker genes placed *T. parvatiensis* at an outlier position with respect to *T. thermophilus* group with a strong bootstrap support (100%) (Figure 2). Correlations based on gene distances scored on the basis of ANI scores, placed *T. parvatiensis* along with SG0.5JP17-16. However, these two strains did not fall into the *T. thermophilus* clade, but clustered with strain CCB_US3_UF1 and *T. igniterrae* instead (Figure 2). The novel species status of strain RL was also reflected in its ANI scores with *T. thermophilus* members (95.03–95.57%), which fall on the borderline for species delineation based on ANI cut-off (95–96%) (Konstantinidis and Tiedje, 2005) (Supplementary Table 2). This is in contrast to the high ANI scores among members of the *T. thermophilus* group (>96%). Tetra-nucleotide based correlations also placed *T. parvatiensis* as an outlier, lying just outside the tight *T. thermophilus* group (Figure 2). The same was reflected in the dendrogram based on pan genes presence-absence (1/0) matrix (Figure 2). Digital DDH values (Supplementary Table 3) were able to separate *T. parvatiensis* from *T. thermophilus* clearly. *T. parvatiensis* vs. *T. thermophilus* dDDH values were in the range 61.0–64.6%, which were below the 70% DDH cut-off for species delineation. This was in contrast to the high intra-species scores among the *T. thermophilus* group (68.9–89%).

Above analyses suggest that species diversification for the genus *Thermus* has taken place by acquisition/deletion/rearrangement of regions which cannot be reflected in the 16S rRNA gene. Conserved genes (31 and 400) are better able to reflect the phylogenetic relationships among the members. However, a drawback of the above methods is that they fall short of taking in account the intragenomic heterogeneity that is quite high among *Thermus* members due to extensive gene shuffling. For this reason, whole genome based methods such as ANI, tetranucleotide frequency and DDH values should be regarded as more accurate phylogenomic methods for estimating phylogenetic relationships within this genus. We have been able to resolve the phylogeny of *T. parvatiensis* by whole genome methods. In spite of having a high percentage similarity with the *T. thermophilus* group, based on 16S rRNA gene sequences, *T. parvatiensis* represents a different species based on whole genome methods, which are more reliable as compared to gene based methods. Thus, *T. parvatiensis*, in course of evolution, has accumulated genome wide differences that have led to its bifurcation with the *T. thermophilus* group, and represents a genetically unique species.

### Analysis of genome flexibility

#### Genome organization

All species belonging to the genus *Thermus*, as described in this study, have been isolated from thermophilic environments (mostly hot spring waters) from all over the world, i.e., from USA (Murugapiran et al., 2013), Japan (Henne et al., 2004), India (Dwivedi et al., 2012), South Africa (Gounder et al., 2011), China (Yu et al., 2015; Zhou et al., 2016), Malaysia (Teh et al., 2012), New Zealand (Hudson et al., 1987; Mefferd et al., 2016), and Iceland (Chung et al., 2000). This illustrates that the genus is spread across the globe, thriving at the most extreme environments. Being confined to stressed niches, these G+C rich (64.8–69.4%) organisms possess small genomes, ranging from 2.01 Mb of *T. parvatiensis* to 2.5 Mb of *T. tengchongensis* YIM 77401 (mean genome size: 2.25 Mb). Considering the powerful evolutionary forces that have been constantly shaping their genomes, in geographically diverse niches, the genome size of *Thermus* shows low variability (Table 1). Already known to shed and rearrange genes that are not uniquely essential for survival, the members of the genus have been successful in maintaining their genome sizes close to the average genome size of the genus. A large part of the genomes is comprised of genomic islands, varying from 0 to 5.85% (Supplementary Table 4).

**Figure 3:**
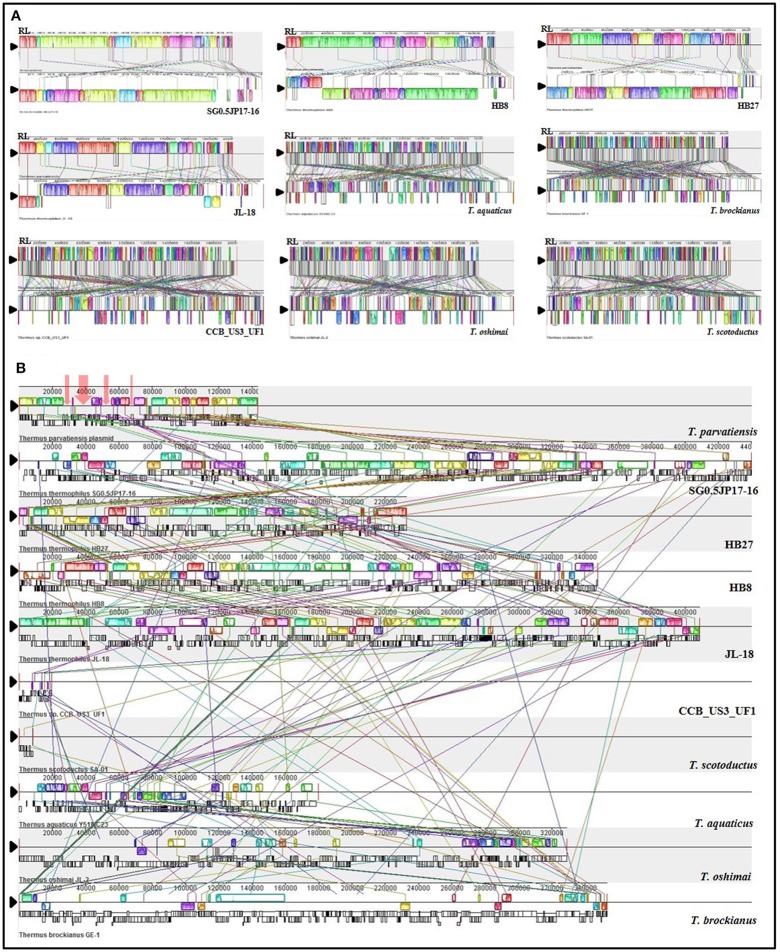
Organizational (synteny) comparisons of *T. parvatiensis* with nine representatives of *Thermus*. Only members with complete genomes have been considered for this analysis. Synteny maps showing **(A)** comparison of the chromosome of *T. parvatiensis* against the chromosomes of other genomes; and **(B)** comparison of plasmid pTP143 against plasmid sequences (concatenated wherever number of plasmids >1) of other *Thermus* members. Boxes of different colors represent locally collinear blocks (LCBs) (or locally conserved regions) connected via lines of the same color to their corresponding positions on other genomes. For each genome, the LCBs above and below the reference line (indicated by black triangle) denote the orientation of the LCBs with respect to the reference sequence (LCBs below the black reference line denote inversions). Black lines (in **B**) below LCBs represent the position of coding sequences. Red arrows on plasmid pTP143 (in **B**) mark the regions that could not be mapped on other *Thermus* plasmids.

To demonstrate the extent of genomic shuffling, synteny maps of the 10 complete genomes were generated (Figure 3). *T. parvatiensis* and SG0.5JP17-16 revealed a conserved organizational synteny with each other and lack of inversions and rearrangements. Synteny was conserved among members of *T. thermophilus* group, however, large blocks of inversions were observed in relation to strains CCB_US3_UF1, *T. scotoductus, T. aquaticus, T. oshimai*, and *T. brockianus*. These demonstrate the huge genome wide rearrangements occurring at the genus level (Figure 3). The same observation was reflected in dot-plot comparisons of *T. parvatiensis* with other members (Supplementary Figure 2).

**Figure 4:**
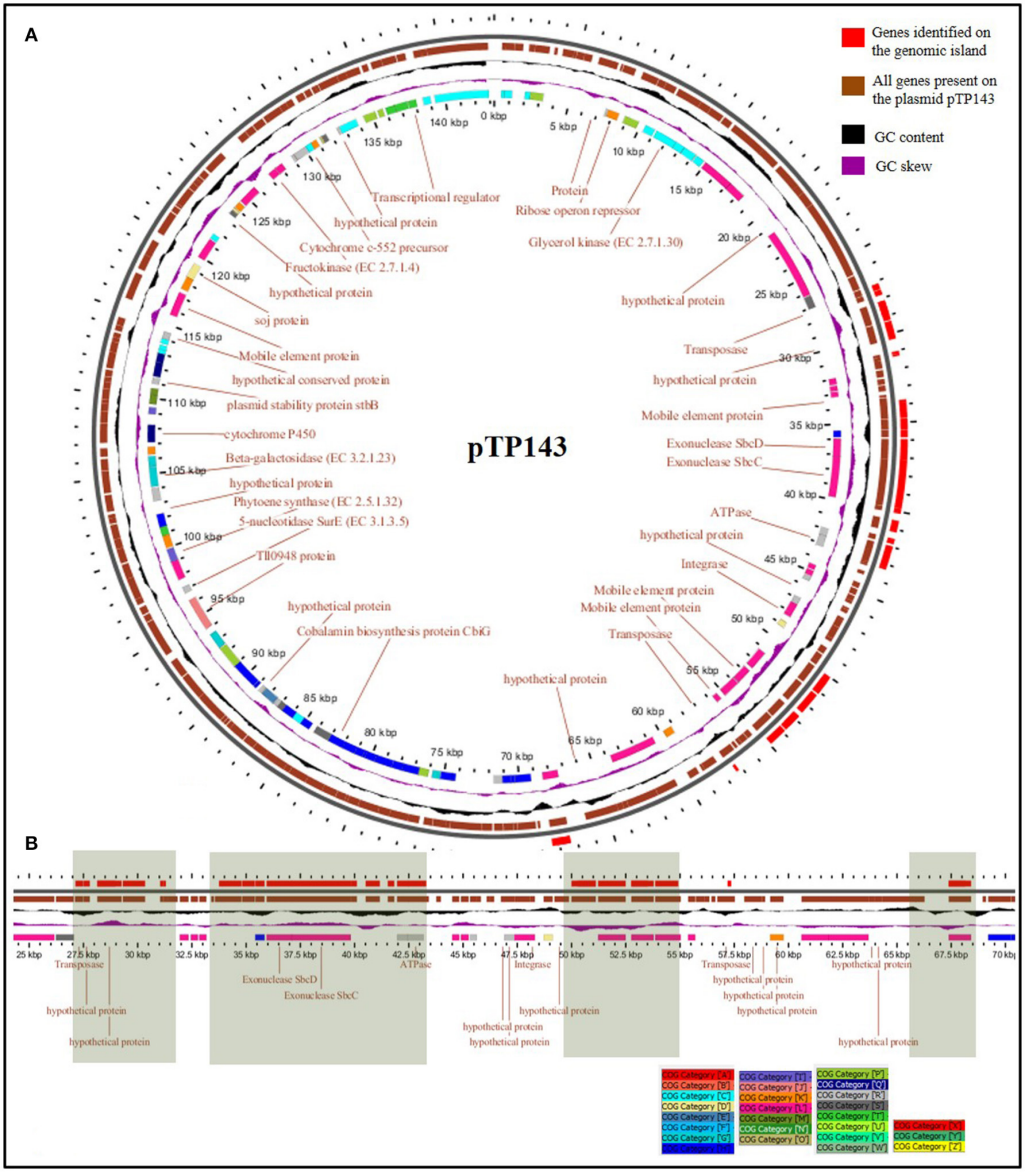
**(A)** Detailed map of plasmid pTP143 with genes assigned to COG categories and depicted in a color-coded manner. Rings from outside to inside represent positions of genomic islands (GI) (red); coding sequences (brown); GC content (black); GC skew (purple) and COG category assignment (multiple colors according to the color key) **(B)** GIs found on the megaplasmid pTP143 (linear view) have been highlighted. These regions contain hypothetical protein coding genes, some plasmid specific genes, a toxin-antitoxin system and repair genes. Most of the genes on the GIs were placed into COG category “L” (replication and repair).

The genes on *Thermus* plasmids are known to undergo vast rearrangement events. In some cases, they have been observed to shift from the plasmid to the chromosome and get stabilized there. In other cases, large plasmids with a majority of non-essential but potentially benefit imparting clusters have been discerned. The former case has been observed in *T. scotoductus* and CCB_US3_UF1, both of which have small plasmids as most of the genes got incorporated on the chromosomes, leaving plasmids with diminished configurations. pTSC8 (8,383 bp), for example, has discarded many non-essential genes like cobalamine biosynthesis pathway genes, plasmid stability genes and chromosome partitioning genes, but retained genes for aerobic and anaerobic respiration to attain a much more compact conformation (Gounder et al., 2011). The latter situation has been observed for plasmid pVV8 (81,151 bp) which was found to be enriched in phosphonate metabolism genes, which are not of common occurrence in *Thermus* genomes (Ohtani et al., 2012). To evaluate this trend in pTP143 and other plasmids, we surveyed genetic clusters commonly observed on *Thermus* plasmids. We observed the presence of advantageous traits such as cobalamin biosynthesis on pTP143 and pTHEOS01 (*T. oshimai* plasmid) and nitrate reduction on pTP143, pTHEOS01 and *T. thermophilus* JL-18 plasmid. To assess possibly laterally acquired regions on pTP143, we performed a comparative mapping of plasmids based on BLASTn comparisons (identity cut-off: 80, coverage cut-off: 80, e-value: 1e-30) across all the *Thermus* plasmids. The plasmid pTP143 of *T. parvatiensis* showed highest identity to the plasmids pTHTHE1601 (identity: 99.3%, query cover: 79%), pTTJL1801 (identity: 97.5%; query cover: 78%), pTT27 of HB27 (identity: 99.0%; query cover: 47%) and pTT27 of HB8 (identity: 98.9%; query cover: 49%). A region on pTP143 that failed to show any homologous regions with any of the plasmids was analyzed as a putatively horizontally transferred region (Supplementary Table 5). This region harbored 3 genes for mobile element proteins, 9 hypothetical protein coding genes, *sbcC* and *sbcD* (hairpin structure resolving nucleases) (Figure 4). The genes present on this locus were identified using BLASTp (identity cut-off: 70%, e-value cut-off: 1.00E-15) on the chromosomes of *T. aquaticus, T. brockianus*, HB8, SG0.5JP17-16, JL-18, CCB_US3_UF1 and even on the chromosome of *T. parvatiensis*.

The genes on the plasmids of *Thermus* are under movement and involved in shifting from the plasmid to the chromosome, in order to get stabilized. In case of *Thermus* species, plasmids perhaps contribute more to the flexibility of genome by either acquiring or shedding of the genes. Incoming genes/pathways may first be incorporated on the plasmid and later be stabilized either on the plasmid itself or through integration into the chromosome. The presence of a genomic island on plasmid pTP143 with multiple mobile elements suggests that it might get mobilized soon. In the process, some other genes may also get shifted from the plasmid to the chromosome. Across *Thermus* genomes, this process has led to either stabilization of the megaplasmid with megaplasmids acquiring a vast majority of genes (SG0.5JP17-16), or it has led to streamlining of the plasmid (*T. scotoductus*).

#### Conserved and variable gene repertoire of *Thermus* group

**Figure 5:**
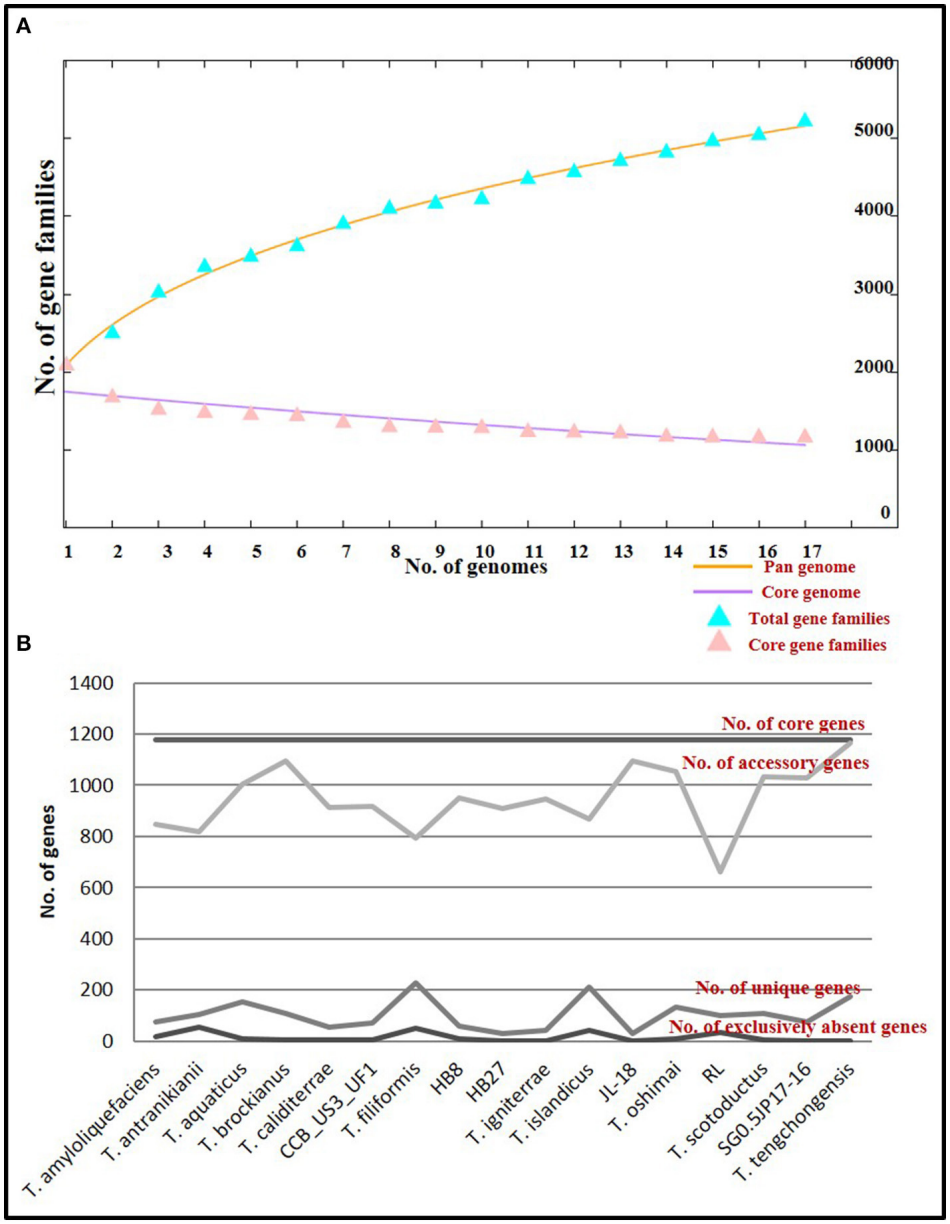
Core and pan genome analysis of *Thermus* genus **(A)** Plot of pan and core genome. The plot represents a stabilized core structure but an open pan-genome; **(B)** Graph showing the number of core genes, accessory genes, unique genes and exclusively absent genes in all genomes under study.

The core genome denotes the conserved functions, whereas, the pan genome denotes the entire genetic potential of a group (Tettelin et al., 2005). The total number of genes constituting the core genome for the genus *Thermus* were 1177. Functional annotation of genes constituting the core genome placed a high number of genes into categories coding for amino acid metabolism and transport (12.15%), translation (10.07%), energy production and conservation (7.75%) and coenzyme metabolism (6.07%), designating these as the conserved functions specific for the genus. The pan genome was estimated at 5188 and constituted accessory genes and unique genes (singletons). Accessory genes are the ones whose orthologs are present in two or more genomes, but not all the genomes. Singletons are genes that are unique to just one genome out of all those compared. The variability in accessory genome depicts the flexibility of the genome structure. Accessory gene number varied from 661 to 1,166 (mean: 946). High number of accessory genes were observed in *T. tengchongensis* (1,166), *T. brockianus* (1,094), JL-18 (1093), *T. oshimai* (1,053), *T. scotoductus* (1,032), SG0.5JP17-16 (1027), and *T. aquaticus* (1003) (Figure 5B). Variability in the number of unique genes (singletons) was observed from 30 to 229 genes (mean 98). A large number of singletons were identified in *T. filiformis* (229), *T. islandicus* (212), *T. tengchongensis* (175), and *T. aquaticus* (154) (Figure 5B). Out of the genomes having high number of accessory genes (higher than mean), 55.5% of the genomes had high number of singletons too. These genomes were *T. aquaticus, T. brockianus, T. oshimai, T. scotoductus*, and *T. tengchongensis*. The accessory and unique components of the genus were enriched in genes belonging to carbohydrate metabolism and transport (8.17 and 8.76% respectively), replication and repair (8.17 and 8.76% respectively), inorganic ion transport and metabolism (5.85 and 5.69% respectively) and signal transduction (5.71 and 5.40% respectively) which reflect the diverse functional counterparts harbored by these organisms. The pan genome of the genus was estimated as an “open” pan genome because a plateau was not observed (Figure 5A) after addition of all genomes to the pan genome plot. Addition of more genomes to the group will lead to expansion of the pan repertoire (Rouli et al., 2015). The genus has thus maintained a conserved core genome, but an expansive and sundry pan genome. The flexibility of the genomes is explained by the high influx of genes into these organisms via horizontal gene transfer. Although, a number of features might get recruited and incorporated in the genome, only those that have a survival benefit for the organism will be retained and the rest will be discarded by the highly active rearrangement events that are continually taking place in these genomes. In due course of time, extensive rearrangement events have led to the establishment of those genomic features that have benefitted the organism for better survival; other dispensable elements were either shed off or transferred to the plasmid. Overall, genome-wide differences and anticipation in accordance with specific gene repertoire required at a niche (niche specialization) can be considered to be driving forces in the evolution of the genus *Thermus*.

**Figure 6:**
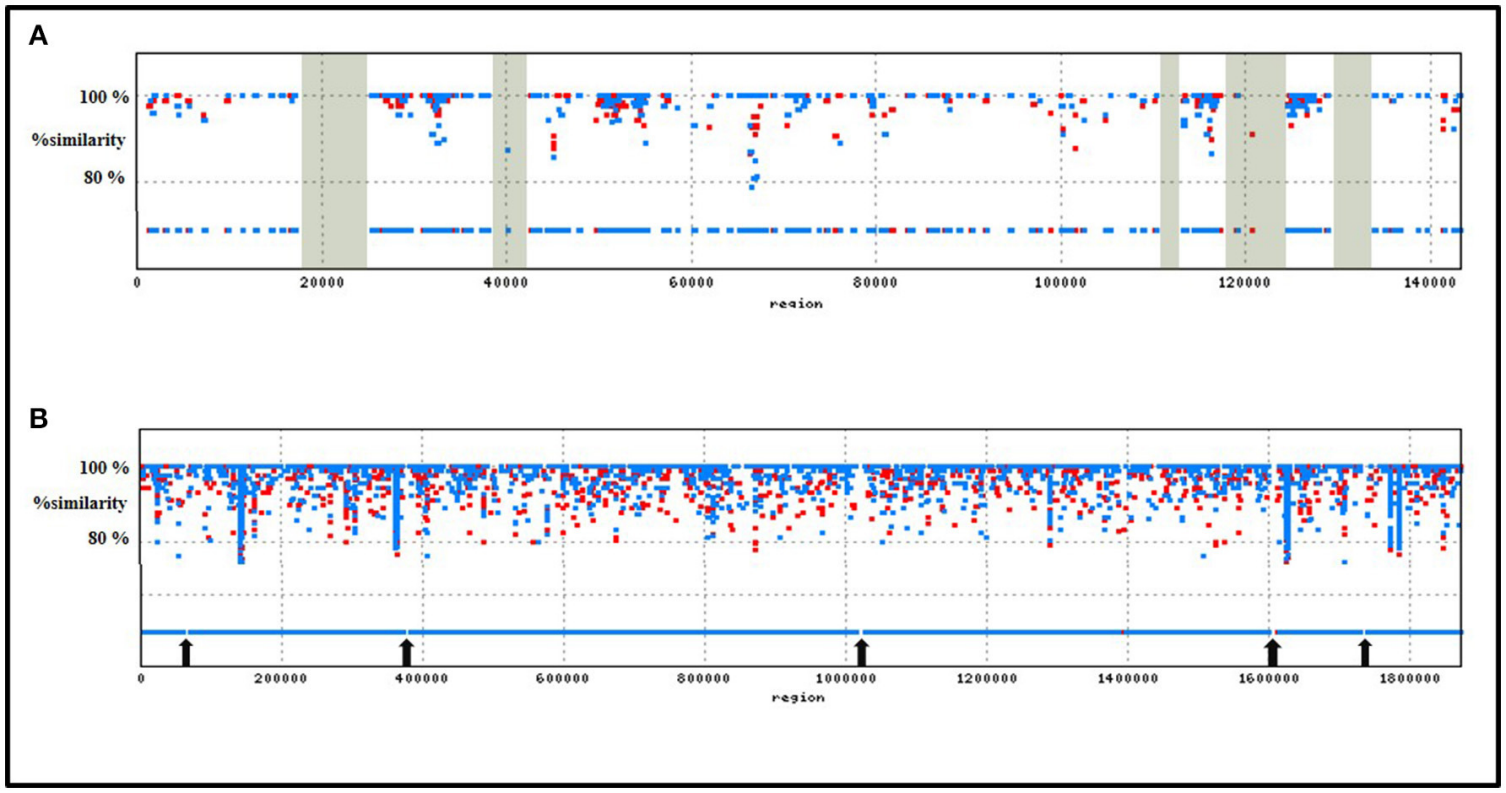
Depiction of metagenomic islands recovered by recruitment of raw reads obtained from the metagenomic sequencing of hot spring water (Manikaran, India) onto *T. parvatiensis* plasmid **(A)** and chromosome **(B)**. MGIs on the plasmid pTP143 (5 MGIs) are highlighted in gray; MGIs on the chromosome of *T. parvatiensis* (5 MGIs) are marked with arrows. Reads mapped to the reference (identity ≥ 80%) are represented as blue (MNW1) and red (MNW2) dots.

In order to designate strain specific, potentially utilizable attributes of *T. parvatiensis*, we identified MGIs of *T. parvatiensis*. The term MGI encompasses those regions of the genome that are identified by mapping metagenomic data from an environment against the genome of an organism isolated from the same niche (Pašić et al., 2009). These regions stand out as “gaps” with little or no reads corresponding to these regions, thus highlighting the strain specific potential that these organisms have accumulated in contrast to the environmental counterparts. Such an analysis was performed for *T. parvatiensis* by aligning raw metagenomic data from hot spring water (Manikaran, India) onto the replicons (Figure 6). *T. parvatiensis* chromosome showed a relatively high coverage of reads (4 ×) with five regions with no coverage of reads (MGIs) (Figure 6). *T. parvatiensis* plasmid pTP143, however had thin coverage of reads (1.9 ×) and five MGIs. Thus, plasmid pTP143 seems to have accumulated more strain specific variations which denote high flexibility of the plasmid. This data further states that the chromosome in case of *Thermus* is more or less stable in terms of the genes it harbors, however, much influx and rearrangements occur via plasmid. The chromosomal MGIs harbored genes for arginine biosynthesis, iron-sulfur cluster assembly proteins, transcriptional regulators, two component system genes and hypothetical genes (Supplementary Table 6). A MGI (4,811 bp) on the plasmid was found to harbor genes specifically involved in environmental response to stress in general and oxidative stress in particular. These genes included radical SAM (S-adenosylmethionine) domain heme biosynthesis protein (heme is a co-factor for hemoproteins that functions in electron transport chain), cytochrome c552 which is particularly involved in electron transport at low aeration, peptide methionine sulfoxide reductase (*MsrA*) known to protect against reactive oxygen and nitrogen species (Weissbach et al., 2002). Cobalamin biosynthesis genes were largely detected on the plasmid MGIs, including uroporphyrinogen-III methyltransferase, *BluB*, adenosylcobalamine-phosphate synthase and *CbiG*. Other prominently represented genes included plasmid stability genes (*ParB, Soj*, and *StbB*) and DNA repair genes (reverse gyrase, *sbcC* and *sbcD*). These genes implicate the conservation of low aeration oxidation response, plasmid stability and DNA repair as strain specific features. In order to get insights about the prevalence of these specialized regions in other genomes, we performed BLASTn (identity > 95%, query coverage > 95%, e-value <1.00E-30) of MGI regions with other *Thermus* genomes. Whereas, genes prevalent on MGI1 of the chromosome did not show significant identity with other members of the genus, MGI2-5 of the chromosome were found to have homologous counterparts in JL-18, SG0.5JP17-16, HB27, and *T. brockianus*. Plasmid MGIs 1, 3, 4, and 5, similarly could be identified on JL-18, SG0.5JP17-16, HB8, HB27, however, plasmid MGI2 of pTP143 was unique in this respect and significant similarity could not be observed with other members. The genes thus annotated on MGIs, were conserved within the *T. thermophilus* group indicating close adaptive relatedness. Chromosomal MGI1 and plasmid MGI2 were identified as strain specific MGIs for *T. parvatiensis*. These attributes suggest the conservation of features that are not directly implicated with the niche, but are retained in the genome as anticipated survival benefits.

### Genus specific survival strategies

#### Abundance of CRISPR arrays

**Figure 7:**
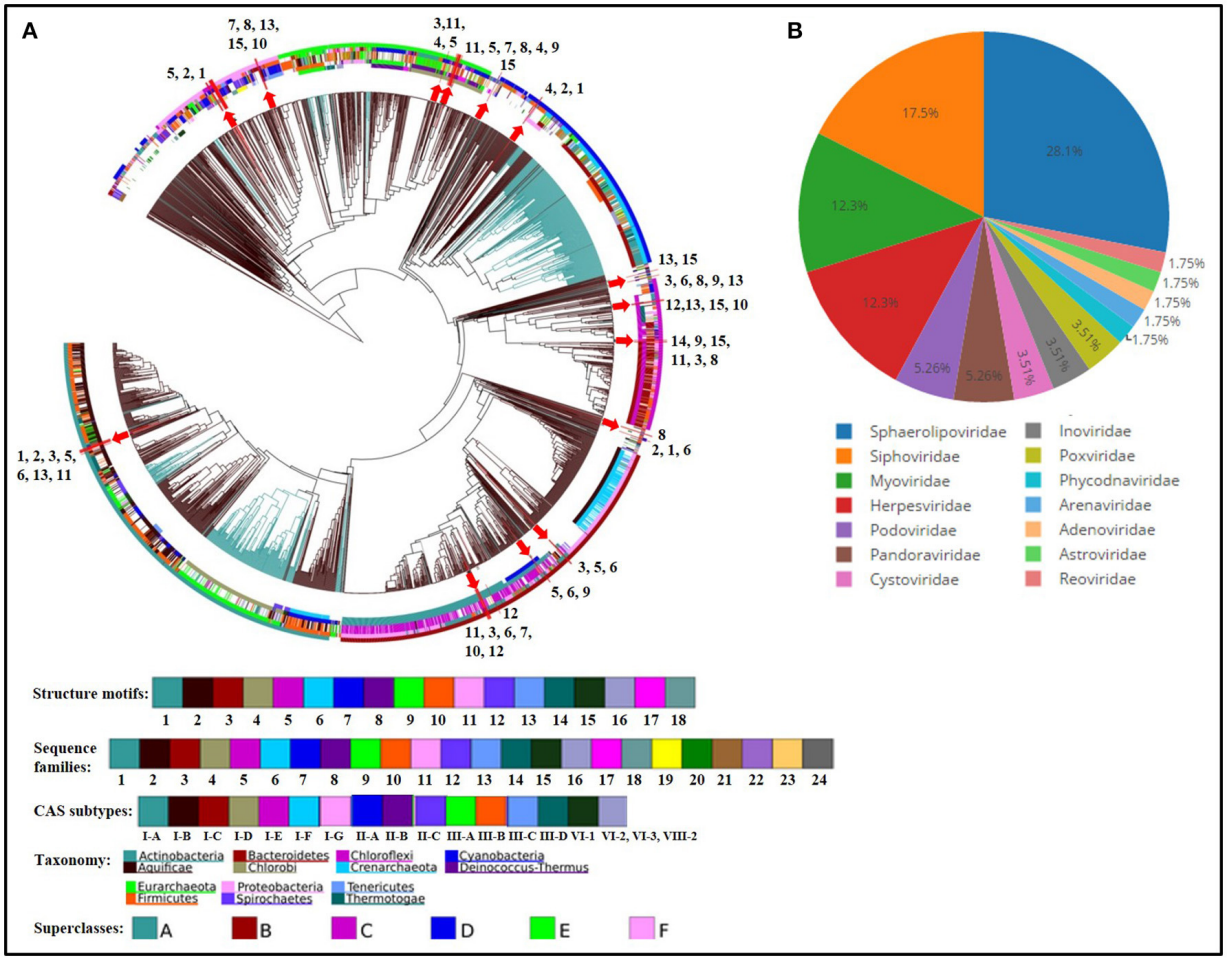
CRISPR repeat **(A)** and spacer **(B)** analyses. **(A)** Dendrogram depicting clustering of CRISPR array repeats from all genomes of *Thermus* spp. against CRISPR repeats database (4,719 consensus repeats). The classification of repeats is based upon the division of all repeats into 24 conserved sequence families and 18 conserved structural motifs. The position of branches representing repeats from *Thermus* spp. is marked by red arrows and depicted by a number which corresponds to a genome, as follows: 1. *T. aquaticus*, 2. *T. amyloliquefaciens*, 3. *T. brockianus*, 4. *T. caliditerrae*, 5. *Thermus* sp. CCB_US3_UF1, 6. *T. filiformis*, 7. *T. thermophilus* HB8, 8. *T. thermophilus* HB27, 9. *T. igniterrae*, 10. *T. thermophilus* JL-18, 11. *T. oshimai*, 12. *T. scotoductus*, 13. *T. tengchongensis*, 14. *T. parvatiensis*, 15. *T. thermophilus* SG0.5JP17-16. Concentric circles are color coded and represent the following from inside out respectively: Structure motifs, sequence families, CAS subtypes, taxonomic groups, superclasses. Black and blue color branches represent bacterial and archaeal sequences respectively. **(B)** Pie diagram representing the relative abundance of viral families infecting *Thermus* genomes, predicted on the basis of CRISPR spacer analysis.

Analysis of CRISPRs among *Thermus* species was performed to get insights into prevalence of viral defense system within the genus. All genomes harbored CRISPR loci, except *T. antranikianii*. Nine CRISPR arrays were identified in *T. filiformis*, HB8, HB27 and *T. igniterrae*, followed by *T. aquaticus* which had 7 CRISPR arrays (Table 3). *T. parvatiensis*, on the other hand, was found to harbor only 1 true CRISPR array. One questionable array was detected in *T. islandicus*. The CRISPR cassette/array number varied from 0-9 with DR length variation from 25 to 37 wide all genomes analyzed (mean CRISPR array count per genome: 5.529). The number of spacers harbored within CRISPR arrays denote the frequency of viral invasions. *T. oshimai* was found to carry the highest number of spacers (134 spacers). A high frequency of viral attacks in these thermophiles was indicated by a high mean number of spacers harbored by each genome (mean: 72.7 spacers per genome). Five *Thermus* genomes (29.4%) harbored >100 CRISPR spacers. Within *T. oshimai* itself, two CRISPR arrays harboring large number of spacers were uncovered. One of the CRISPR arrays harbored 88 copies of a single DR consensus (CGGTCCATCCCCACGGGCGTGGGGACTAC; DR length: 29 bp), with an equally high number of spacers. The other array (DR consensus = CTTTGAACCGTACCTATAAGGGTTTGAAAC; DR length: 30 bp) had acquired 67 spacers. On the contrary, within the same genus, an array containing only 3 spacers was also observed. This indicates high variation among CRISPR elements within the species. *Cas* genes were extracted from true CRISPRs and annotated (Table 3). The Cas system in *Thermus* was found to be composed of genes belonging to types I, III, and IV (Supplementary Figure 3). Universal type genes *cas1* and *cas2* were identified on CRISPR loci. Apart from this, the class I effector cas3 which is responsible for the helicase and DNase activity was also annotated (Table 3). Thus, cleavage of foreign entities entering *Thermus* genomes is brought about by type I, III, and IV mediated action. Analysis of repeats based on sequence and structure (Figure 7) was performed. Sequence families 1 and 18 were predominant (spotted in 7 and 6 genomes respectively) (Supplementary Table 8). Among structural motifs (based on the classification of motifs into 33 groups), motifs 25 and 5 were most represented (8 and 6 genomes respectively). Motif 6, motif 20, motif 23, and motif 31 were least represented (1 genome each) (Supplementary Tables 7, 8). Additionally, on the basis of repeat-cas binding projections, probable *cas* genes harbored by *Thermus* genomes were predicted and found to belong to types I, III, and IV. Separate cluster trees were constructed for structure motifs (based on the classification of structural motifs into 18 groups) to denote the placement of *Thermus* repeats among all consensus repeats present in the database. A majority of repeats (10 repeats) mapped onto motif 1 and occupied a close phylogenetic position within the cluster tree (Supplementary Figure 4). In order to discern the phages most frequently attacking *Thermus* genomes, we analyzed spacer matches with viral sequence database (Supplementary Table 9). Positive inferences were based only on the results that satisfied the stringency criteria. A number of phages of different families were detected to infect *Thermus* species (Figure 7B). Among these, phages of families *Sphaerolipoviridae, Siphoviridae, Myoviridae*, and *Herpesviridae* were identified as the most prominent bacteriophages. Earlier *Siphoviridae, Myoviridae, Inoviridae*, and *Tectiviridae* have been reported from *Thermus* species (Yu et al., 2006). However, our analysis revealed the dominance of *Sphaerolipoviridae* (28.1%), with known thermophilic phages P23-77 and IN93 being prominently detected. A detailed list of viruses, the invasion memory of which is incorporated within *Thermus* CRISPRs is mentioned in Supplementary Table 9. Our analysis thus reveals the abundant presence of defense mechanism and frequent viral encounters in this genus. Even though a large number of spacers (1,223) were analyzed, only 62 (5.07%) could be assigned significant matches to the viral database.

**Table 3:** Summary of CRISPR elements found across all *Thermus* genomes under this study.

**Species**	**Confirmed CRISPRs**	**DR length**	**Spacers**	***Cas* types**	***Cas* genes**
*T. parvatiensis*	1	37	9	Other	*cas6, cas2*
*T. thermophilus* HB27	9	32-37	6+3+15+7+7+6+6+13+9 = 72	I, III, IV	*csa3, cas2, cas10, csm2gr11, csm3gr7, csx1, cmr3gr5, cmr4gr7, cmr5gr11, DinG, cas5, cas8c, cas7b, cas1, cas4, WYL, cas8b5, cas7, cas3, cas6*
*T. thermophilus* HB8	9	29-37	14+4+3+12+9+12+23+20+12 = 109	I, III	*csx1, cas1, cas2, cas10, csm2gr11, csm3gr7, csm3gr5, cmr4gr7, cmr5gr11, WYL, cas3HD, cas8e, cse2gr11, cas7, cas5, cas6e, cas1, cas8b5, cas3, cas6*
*T. thermophilus* JL-18	6	29-37	21+23+7+25+19+5 = 100	I	*WYL, cas3HD, cas8e, cse2gr11, cas7, cas5, cas6e, cas1, cas2, cas4, cas8b5, cas3, cas6, csa3, cas8U1, csb2gr5, casR*
*T. thermophilus* SG0.5JP17-16	6	28-37	3+13+6+10+6+18 = 56	I	*casR, cas7, csb2gr5, cas3, cas8U1, cas4, csa3, cas2, cas1, WYL, cas8b5, cas5, cas6, cas8c, cas7b*
*T. scotoductus*	3	29-30	9+36+42 = 87	I	*cas2, cas1, cas6e, cas5, cas7, cse2gr11, cas8e, cas3HD, WYL*
*T. oshimai*	5	29-36	14+6+88+30+4 = 134	I, III, IV	*cas6, csx1, csm3gr7, csm4gr5, csm2gr11, cas10, cas2, csx1, WYL, cas3HD, cas8e, cse2gr11, cas7, cas6e, cas1, csa3, DinG, cas3*
*T*. sp. CCB_US3_UF1	7	28-36	3+17+14+23+18+9+12 = 96	I, III	*csm3gr7, csm5gr11, cmr4gr7, cmr3gr5, cas10, csx1, cas4, cas3HD, cas6, cas8b1, cas7, csm2gr11, csm4gr5, cas1*
*T. aquaticus*	7	26-36	7+5+22+3+6+12+3 = 58	I, III	*cas2, cas1, csx1, csa3, cas5, cas7, cas8b1, cas3HD, cas4, cas10, csm2gr11, csm3gr7, csm4gr5, cas6, csm5gr11, csm4gr7*
*T. brockianus*	8	29-37	9+14+7+10+12+8+25+27 = 112	I, III	*cas6, cas10, csm2gr11, csm3gr7, csm4gr5, csx1, cas2, cas1, cmr3gr5, cmr4gr7, cmr5gr11, cas1, cas4, cas3, cas3HD, cas8b1, cas7b, cas5, cas8c, cas8e, cse2gr11, cas7, cas5, cas6E*
*T. antranikianii*	0	0	0	—	—
*T. filiformis*	9	25-37	3+24+4+5+6+22+5+12+7 = 88	I, III, IV	*cas4, WYL, DinG, cas3HD, cas8e, cse2gr11, cas7, cas5, cas6e, csx1, cas10, cmr3gr5, csm3gr7, cmr4gr7, cmr4gr11, cas2, cas6, cmr2gr11, csm4gr5, casR, cas2, cas1, cas3D, cas8b1, cas7b*
*T. islandicus*	0	0	0	I, III	*cas1, csx1, cas6, cas3, cas7, cas8b5, WYL, cas8b1, cas7b, cas5, cas2, csa3, csm3gr7, csm4gr5, csm2gr11, cas10*
*T. igniterrae*	9	28-36	5+23+3+11+3+4+15+17+28 = 109	I, III	*csm3gr7, csm4gr5, csm2gr11, cas10, csx1, WYL, cas3HD, cas8e, cse2gr11, cas7, cas5, cas6e, cas1*
*T. caliditerrae*	4	35-36	21+18+9+10 = 58	I, III	*csm3gr7, cmr5gr11, cmr3gr5, cas10, cas6, csx1, csm2gr11, csm4gr5, cas2, cas1, cas3*
*T. amyloliquefaciens*	5	29-36	15+5+9+6+18 = 53	I, III	*csx1, csm4gr5, csm3gr7, csm2gr11, cas10, cas6, csx1, cas2, csa3, cas1, cas6e, cas5, cas7, cse2gr11, cas8e, cas3HD, WYL*
*T. tengchongensis*	4	30-37	67+3+12+9 = 91	I, III	*csa3, csm2gr11, csa3, csa3, casR, cas7, cas8b1, cas3HD, cas4, cas3, cas5, cas8b5, WYL*

CRISPRs constitute the characteristic prokaryotic and archaeal adaptive as well as inheritable immune system composed of short repeat sequences (direct repeats/DRs) interspaced with short segments of nucleotides known as spacers. Spacers represent the memory of past invasions by foreign genetic elements like viruses (Barrangou et al., 2007) or plasmids (Marraffini and Sontheimer, 2008). Spacers are incorporated into CRISPR loci whenever a bacteriophage infects the organism. This way, a CRISPR array can be considered as a library of past viral invasions faced by an organism. CRISPR arrays are associated with CRISPR associated genes (*Cas* genes), which are present in the vicinity of CRISPR arrays. Together with virus specific spacers, *Cas* genes encode an arsenal of proteins and RNAs, which in conjugation, destroy the foreign element, the next time it invades (Mojica et al., 2005; Barrangou et al., 2007). The CRISPR system is thus, a defense mechanism against bacteriophage invasions on the bacterial genome. CRISPR arrays that lack the requisite *Cas* genes in their vicinity are known as questionable or false CRISPRs. Predominance of viruses in thermophilic niches and consequently prevalence of CRISPRs in thermophilic genomes is known (Anderson et al., 2011; Weinberger et al., 2012b). We analyzed the CRISPR loci from all *Thermus* genomes in this study in order to investigate the probable viruses infecting the genus and to uncover the organization of *cas* genes. Our examination divulged the ubiquity of CRISPR arrays within the genus *Thermus*, reflecting a resilient viral defense system. The predominance of CRISPRs among the *Thermus* group suggests presence and activity of phages in thermophilic environments. A wide scenario of phage invasion in *T. oshimai* was particularly denoted by high number of spacers present in this species. The uneven distribution of CRISPR arrays within a group can be explained by the hypothesis that probably the cost of harboring CRISPR elements in particular bacteria outweighs the benefits harnessed (Weinberger et al., 2012a). *Cas* genes, which are momentous to the functioning of CRISPRs, are divided into two classes (class I and II), depending on their mechanism of action. Class I CRISPR-Cas systems act by employing a number of Cas proteins which bring about the required action in a cascade of events. Class II systems, on the other hand, rely on single effector proteins for binding and cleavage of the target site. Based on the specific proteins involved, class I is further divided into types I, III and IV and class II is divided into types II and V. Widespread presence of type I (15 genomes) and type III (12 genomes) was observed in *Thermus*. Type IV systems were detected only in 3 genomes. Type IV systems have been known to be rare (<2%) in both bacteria and archaea (Makarova et al., 2015). Evolutionary relationships were uncovered on the basis of repeat sequences by clustering repeat sequences into conserved sequence families and structural motifs (Lange et al., 2013). CRISPR DRs transcribe repeat RNA sequences which serve as Cas protein binding templates. Repeat sequences show significant conservation in their sequence as well as hairpin structure forming motifs (Lange et al., 2013). Sequence conservation has been used as a criterion for clustering of DRs into families. Similarly, structural motif grouping is based on RNA loop structures. Using this analysis, we were able to identify the distribution of sequence families and structural families within this genus. Interestingly, motifs 20 and 31, which were identified in *T. filiformis* and HB8, have been known to constitute a mixture of both bacterial and archaeal domains. Viral diversity analysis delineated most probable viral predators for the *Thermus* group. A high proportion of spacers, however, were left un-assigned, implying the huge viral genosphere that is yet unexplored.

#### Genes imparting competence

**Figure 8:**
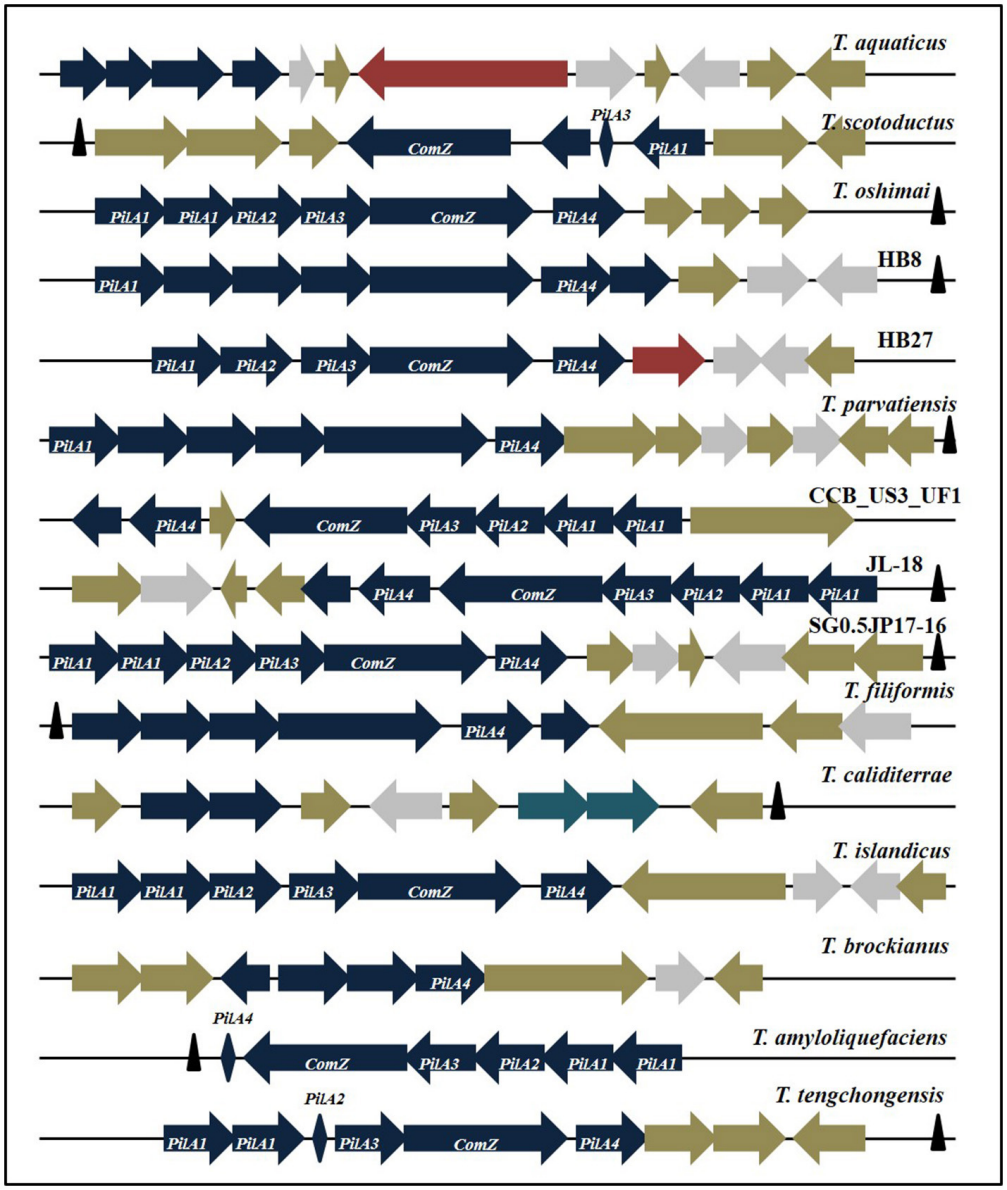
Organization of putatively horizontally transferred *PilA-ComZ* locus genes from all *Thermus* genomes in this study. Blue, Pilus family proteins; Gray, Nucleases; Tan, Hypothetical/Conserved proteins; Maroon, Transposase/Conjugative element; Rhomboid, unformed CDS; Navy blue, Toxin-antitoxin elements; Black triangles, tRNAs.

Genes implicated in natural transformability of HB27 include *PilA1, PilA2, PilA3, PilA4, ComZ, CinA, DprA, ComEA, ComEC, PilF, PilC, PilM-N, PilN-O-W, ComF, PilQ*, and *PilD*. Apart from HB27, all of the above genes were harbored by *T. oshimai*, CCB_US3_UF1, *T. islandicus*, JL-18, and SG0.5JP17-16. In this study, all *Thermus* genomes were found to show a homogeneous profile with respect to genes except *PilA1-PilA4* and *ComZ*. Among competence genes, *PilA* gene plays a decisive role in efficient translocation (Schwarzenlander et al., 2009). *PilA1-A4* genes and *ComZ* are present as a cluster in all genomes, however the arrangement and organization of genes show a difference across genotypes. The *PilA-ComZ* locus was found to be harbored in all *Thermus* genomes except *T. igniterrae* and *T. antranikianii*. Either these strains have not yet acquired the cluster or the cluster has been missed out during sequencing (draft genomes). The *PilA-ComZ* operon contains *PilA1, A2, A3, A4*, and *ComZ* as principal genes. The operon, however, also contains other genes with pilin/putative pilin/pseudopilin domains and genes coding for hypothetical proteins. The *PilA-ComZ* locus in *Thermus* was found to be comprised of 9–12 genes out of which 4–7 genes were pseudopilins with significant similarity to the *PilA* genes of strain HB27 (Figure 8). Apart from pseudopilin genes, the locus is comprised of genes coding for hypothetical proteins, chromosome segregation protein, apolipoprotein and DUF820 superfamily nucleases. *PilA1* gene (present in 14 genomes) was found to be duplicated in 7 genomes. The two copies of *PilA1* were, however, not identical and showed identity ranging from 67 to 81%. By convention, we have named the *PilA1* of HB27 (471 bp) as copy 1. Copy 2 of *PilA1* (present in 7 strains) is a smaller gene ranging from 272 to 373 bp (mean = 329 bp). In *T. caliditerrae*, only a fragmented copy (135 bp) of *PilA1* was recovered. *PilA2* (582–614 bp) was recovered in 7 strains as a complete copy. In case of *T. scotoductus*, identity at the C- and N- terminals and non-identity at the middle of the gene sequence was observed. A truncated version of *PilA2* was identified in *T. tengchongensis. PilA3* was recovered in a complete state (685–746 bp) in nine strains. In *T. scotoductus*, a truncated *PilA3* was detected. The most deviant forms among *PilA* genes were observed for *PilA4*. Patterns of alignment among all *PilA* genes best denote this observation (Supplementary Figure 5). The alignment features are most consistent (>70%) in case of *PilA1* through *PilA3* genes. In case of *PilA4*, similarity among genes is observed only at the C- and N- termini of the genes. *PilA4* of HB27 is 396 bp in size, however, in the other 13 strains harboring *PilA4*, only a small part (87–121 bp) showed significant similarity (>80%, e-value: 1E-15) to *PilA4* of HB27. Additionally, truncated versions of *PilA4* were observed in *T. aquaticus, T. caliditerrae*, and *T. amyloliquefaciens*.

A complete *PilA-ComZ* locus was identified in HB27, *T. oshimai*, CCB_US3_UF1, JL-18, SG0.5JP17-16, and *T. islandicus*. However, in other genomes, we could identify the presence of “genetic scars.” Genetic scars are truncated genes or pseudogenes (without start/stop codons), but show significant identity to functional *PilA* gene regions of HB27 (taken as a reference for all comparisons here). Two genetic scars were identified in *T. scotoductus* (*A3* and *A2*), two in *T. caliditerrae* (*A1* and *A4*), one in *T. amyloliquefaciens* (*A4*), one in *T. aquaticus* (*A4*) and one in *T. tengchongensis* (*A2*). Interestingly, an IS4 family transposase, which is known to be found widely across the *Thermus* genomes was found incorporated in the *PilA*-*ComZ* locus of *T. aquaticus*. This can be an evidence for recent transposition activity at the locus. Another evidence is the presence of a conjugative protein in the *PilA*-*ComZ* locus of HB27. Interestingly, a toxin-antitoxin (TA) gene system was found to be incorporated into the competence locus of *T. caliditerrae* and showed similarity to a TA pair in *Rhodothermus marinus*. Horizontally transferred regions are generally found to be associated with tRNA genes (Darmon and Leach, 2014). As expected, tRNA genes were located in close proximity of the *PilA-ComZ* loci of 10 genomes in this study. Out of the ten genomes in which tRNA genes were associated with the competence locus, seven are still not fully formed, thus giving a strong boost to the hypothesis of recent horizontal acquisition of the locus. Genetic relatedness of *Thermus* strains on the basis of *PilA1*-*A4* genes was reconstructed to infer the evolutionary history of *PilA* genes on the basis of their sequence development (Supplementary Figure 6). *PilA1* copy1 and copy2 gene trees highlighted strain CCB_US3_UF1 and JL-18 as an outlier for both trees and *T. parvatiensis* as an additional outlier for *PilA1* copy1 gene. CCB_US3_UF1 and JL-18 retained their outlier positions in all other trees. In case of *PilA2* and *PilA3* sequences, *T. scotoductus* emerged as an outlier. For *PilA4, T. parvatiensis*, CCB, *T. scotoductus* and JL-18 lie on the out branches of the dendrogram.

Genes responsible for imparting competence in the genus *Thermus* can be divided into two groups: the first group of genes are responsible for uptake of DNA and the second group is responsible for transport of DNA into the cell. Some of these genes belong to the T4P family of proteins which form a complex on the membrane of the cell, spanning the S-layer, Outer Membrane (OM), Secondary Cell Wall Polymers (SCWP) and Peptidoglycans (PG) which comprise the periplasm and the inner membrane (IM). Sixteen genes have been known to play a role in natural transformation in HB27, the transformation machinery of which is the most extensively studied. PilQ is a secretin which forms a macromolecular homopolymeric complex on the outer membrane (Burkhardt et al., 2011) and binds to the incoming DNA. Once DNA reaches the periplasmic space, the pseudopilus proteins (pilA1 through pilA4) come into play, forming subunits of the DNA translocator complex present in the periplasm. PilA1-PilA4 pilins form a shaft like structure in the periplasm which is attached to the outer membrane through PilQ (Burkhardt et al., 2011) and to the inner membrane through a motor ATPase PilF. PilF uses the energy of ATP hydrolysis to draw DNA toward the inner membrane (Rose et al., 2011; Collins et al., 2013). Double stranded DNA further binds to the inner membrane protein ComEA. An inner membrane channel protein, ComEC (co-transcribed along with ComEA) has recently been shown to modulate the expression of *PilA4* and *PilN* in relation to environmental cues like nutrient limitation and low temperature (Salzer et al., 2016). The DNA passing through the IM is single stranded DNA. Hence, a nuclease has to be acting on double stranded DNA to make it single stranded. This nuclease has not been identified yet.

A stable arrangement of the *PilA-ComZ* locus could be observed in *T. oshimai*, CCB_US3_UF1, JL-18, SG0.5JP17-16, and *T. islandicus*. The *PilA*-*ComZ* locus represents a small horizontally acquired region (genomic islet) on the chromosome. It can be distinguished by high number of hypothetical protein coding genes, gene duplications and their association with tRNA genes (Darmon and Leach, 2014). Gene duplication events observed in case of *PilA1* genes demonstrate species radiation forces and amenability of the genomes to evolutionary forces (Roth et al., 2007). The presence of a transposase in *T. aquaticus*, a conjugative element in HB27, TA element in *T. caliditerrae* and truncated *PilA* genes (genetic scars) on the locus indicate recent horizontal origins. Truncated *PilA4* genes observed across this genus denote that it has either not developed or has undergone degradation, determined by the presence of genetic scars which show similarity to N-/C-terminals of *PilA4* of HB27, but not to the complete gene. The close association of a TA system with *PilA*-*ComZ* cluster of *T. caliditerrae* reflects recent acquisition of this cluster. TA systems have been found to be associated with genomic islands and other mobile elements. Association with a TA system, promotes the maintenance of a horizontally acquired island and stabilization into the host genome (Rowe-Magnus et al., 2003; Iqbal et al., 2015). Generally, horizontally transferred loci are marked with pseudogenes as there is a strong selection pressure against these regions (Hao and Golding, 2010). Pseudogenes are non-functional versions of a previously functional gene, which are in the process of getting lost from the genome (Hao and Golding, 2010). Pseudogenes are known to be associated with recently laterally acquired regions or failed HGT events (Hao and Golding, 2010). The predominance of pseudogenes can be due to the high rates of gene turnover in laterally acquired regions. Some of the truncated genes may get stabilized and some eliminated in due course of time. The evidence thus provided leads us to believe that the competence machinery in *Thermus* is of horizontal origin and in course of evolution, may get stabilized or eliminated. The presence of a highly efficient transformation system however does not ensure the incorporation of incoming DNA into the host genetic material. Natural transformation in native conditions is activated during environmental challenges such as starvation, wherein DNA is taken up from the environment (Seitz and Blokesch, 2013). Most of the nucleic acid taken up during this process is used to fulfill nutritional requirements. In this process, some amount of DNA may get incorporated into the host genome, thus diversifying the host pan repertoire and expanding the already diverse arsenal of *Thermus* group.

#### Choice between natural competence and viral resistance

The pilus structure in *Thermus* imparted by the T4P genes plays a role not only in twitching motility and natural competence, but also in bacteriophage infection. *PilA* mutants have been shown to lose not only twitching motility and natural competence, but are also resistant to phage infection in HB27 and HB8 strains (Tamakoshi et al., 2011). On the basis of our comparative analysis for the *Thermus* group, we propose a link between pilus gene diversification and CRISPR abundance. Our data suggests continued acquisition and evolution of pilus gene structure among the analyzed *Thermus* genomes. Along with this, a CRISPR system with high number of spacers suggests a robust immune machinery against bacteriophages. In case of *T. islandicus*, only one questionable CRISPR array was observed, and no CRISPR array was observed in the case of *T. antranikianii*. Interestingly, a *PilA*-*ComZ* locus was also absent in *T. antranikianii*. The two observations, when coupled together suggest that *T. antranikianii* might be resistant to a huge proportion of viruses due to lack of pilus system genes which are implicated in phage entry, thus avoiding the need for harboring the CRISPR system. Other *Thermus* members have, on the other hand, chosen pilus mediated natural transformation as an important evolutionary trait, even though it makes them more susceptible to phage attacks, leading them to harbor more frequent CRISPR defense systems. Thus, natural transformation may be regarded as an overall benefit imparting trait in the small thermophilic genomes of *Thermus*. Natural transformation has played a role in survival of these organisms since long. Therefore, the dispersal of this system is a rather favorable phenomenon wide this genus even though it imposes an additional cost of harboring CRISPR machinery on them.

## Conclusion

Organisms belonging to the genus *Thermus* have occupied a significant position and have diversified present knowledge about thermophilic survival. *T. parvatiensis*, in accordance with its affiliation to the genus has maintained a small genome and a plastic plasmid. Plasmids of *Thermus* are hotspots for genome dynamism, acting as centers of influx as well as efflux of genes and pathways in this genus. A dynamic pan genome along with strain specific gene reservoirs signify acquisition and conservation of favorable attributes. One of the factors contributing towards this dynamism is an active natural transformation system of this genus. The natural competence machinery in *Thermus* has proved to be an overall advantageous trait for the dual reason of nutrition limitation and genetic variability. It has, however, made these organisms susceptible to viral grazing, leading to the development of viral defense arsenal, known as CRISPRs. The efficacy of choices made has led to proficient sustenance of this genus in the face of adversity and beyond.

## Ethics statement

This article does not contain any studies with human participants or animals performed by any of the authors.

## Author contributions

RL conceived the study and supervised manuscript preparation. CT performed the analysis except CRIPSR analysis. HM performed CRISPR analysis. HK prepared all tables and figures. VD, RN and KK helped in data interpretation and drafting of the manuscript.

### Conflict of interest statement

The authors declare that the research was conducted in the absence of any commercial or financial relationships that could be construed as a potential conflict of interest.
