# Supplementary material for: Complete Genome Analysis of Thermus parvatiensis and Comparative Genomics of Thermus spp. Provide Insights into Genetic Variability and Evolution of Natural Competence as Strategic Survival Attributes
Source: Front Microbiol. 2017 Jul 27;8:1410. doi: 10.3389/fmicb.2017.01410 (PMC5529391; doi:10.3389/fmicb.2017.01410)
Supplement: Supplementary file 1 [file Table1.PDF]

Supplementary table 1: Sequencing data generation summary for *T. parvatiensis* RL.

Sequencing platform	PacBio RS II
Total raw data generated	2.488 GB
Average read length	9,878 nt
Reads generated	224,211
Bases sequenced	857,926,800
Average sequencing depth	428×
