# Supplementary material for: Complete Genome Analysis of Thermus parvatiensis and Comparative Genomics of Thermus spp. Provide Insights into Genetic Variability and Evolution of Natural Competence as Strategic Survival Attributes
Source: Front Microbiol. 2017 Jul 27;8:1410. doi: 10.3389/fmicb.2017.01410 (PMC5529391; doi:10.3389/fmicb.2017.01410)
Supplement: Supplementary file 2 [file Table2.PDF]

Supplemetary table 2: Two-way ANI scores of *Thermus* species with each other. ANI scores among the *T. thermophilus* group are highlighted with blue, whereas those of *T. parvatiensis* with *T. thermophilus* group are marked with pink.

	T. thermophilus HB27	*T. thermophilus* HB8	*T. parvatiensis* RL	*T. thermophilus* SG0.5JP17-16	*T. thermophilus* JL-18	*T. oshimai*	*T. scotoductus*	*T. caliditerrae*	*T. antranikianii*	*T.* sp. CCB_US3_UF1	*T. igniterrae*	*T. islandicus*	*T. aquaticus*	*T. filiformis*	*T. amyloliquefaciens*	*T. tengchongensis*	*T. brockianus*
T. thermophilus HB27	100	98.9	95.04	96.55	96.98	82	81.21	81.81	81.26	82.17	82.75	83.96	83.15	80.92	82.06	81.74	82.52
T. thermophilus HB8	98.9	100	95.03	96.65	96.9	81.87	81.32	81.85	81.2	82.25	82.8	84.01	83.19	80.92	82.03	82.07	82.54
T. parvatiensis RL	95.04	95.03	100	95.57	95.41	81.56	81.14	81.76	80.74	82.3	83.48	83.26	82.87	78.81	82.43	82.06	82.99
T. thermophilus SG0.5JP17-16	96.55	96.65	95.57	100	96.78	81.82	81.61	81.74	81.36	82.14	82.76	83.66	82.83	80.8	82.23	82.37	82.55
T. thermophilus JL-18	96.98	96.9	95.41	96.78	100	82.42	81.42	81.69	81.17	82.13	82.62	83.66	83.25	80.79	81.93	82.36	82.39
T. oshimai	82	81.87	81.56	81.82	82.42	100	80.96	81.44	80.88	82.03	82.21	81.59	82.56	80.59	81.78	81.28	81.59
T. scotoductus	81.21	81.32	81.14	81.61	81.42	80.96	100	84.13	94.76	82.24	82.74	81.23	81.87	79.57	86.67	88.71	82.04
T. caliditerrae	81.81	81.56	81.76	81.74	81.69	80.81	83.99	100	83.67	83.65	84.08	81.7	82.31	77.74	85.55	91.24	82.55
T. antranikianii	81.26	81.2	80.74	81.36	81.17	80.88	94.76	83.67	100	82.19	83.55	81.16	81.6	79.34	86.39	88.21	82.26
T. sp. CCB_US3_UF1	82.17	82.25	82.3	82.14	82.13	82.03	82.24	83.33	82.19	100	84.24	82.1	82.71	80.24	83.17	82.82	83.03
T. igniterrae	82.75	82.8	83.48	82.76	82.62	82.21	82.74	83.68	83.55	84.24	100	82.58	83.27	80.47	83.6	83.47	83.69
T. islandicus	83.96	84.01	83.26	83.66	83.66	81.59	81.23	81.7	81.16	82.1	82.58	100	83.95	80.11	81.71	81.47	82.03
T. aquaticus	83.15	83.19	82.87	82.83	83.25	82.56	81.87	82.31	81.6	82.71	83.27	83.95	100	80.52	82.53	82.1	82.48
T. filiformis	80.92	80.92	78.81	80.8	80.79	80.59	79.57	79.86	79.34	80.24	80.47	80.11	80.52	100	79.95	79.96	80.1
T. amyloliquefaciens	82.06	82.03	82.43	82.23	81.93	81.78	86.67	85.55	86.39	83.17	83.6	81.71	82.53	79.95	100	87.38	82.55
T. tengchongensis	81.74	82.07	82.06	82.37	82.36	81.28	88.71	91.24	88.21	82.82	83.47	81.47	82.1	79.96	87.38	100	82.31
T. brockianus	82.52	82.54	82.99	82.55	82.39	81.59	82.04	82.55	82.26	83.03	83.69	82.03	82.48	80.1	82.55	82.31	100
