# Supplementary material for: Complete Genome Analysis of Thermus parvatiensis and Comparative Genomics of Thermus spp. Provide Insights into Genetic Variability and Evolution of Natural Competence as Strategic Survival Attributes
Source: Front Microbiol. 2017 Jul 27;8:1410. doi: 10.3389/fmicb.2017.01410 (PMC5529391; doi:10.3389/fmicb.2017.01410)
Supplement: Supplementary file 3 [file Table3.PDF]

Supplementary table 3: Digital DDH (dDDH) values calculated for all *Thermus* species against each other using Genome to Genome Distance Calculator (ggdc.dsmz.de)

The dDDH values among *T. thermophilus* group are marked with blue. The dDDH values of *T. parvatiensis* with the closely related *T. thermophilus* group are marked with pink, all below the DDH cutoff (70%).

	T. igniterrae	*T. antranikianii*	*T. caliditerrae*	*T.* sp. CCB_US3_UF1	*T. filiformis*	*T. thermophilus* HB8	*T. thermophilus* HB27	*T. islandicus*	*T. oshimai*	*T. thermophilus* JL-18	*T. parvatiensis*	*T. scotoductus*	*T. thermophilus* SG0.5JP17-16	*T. amyloliquefaciens*	*T. brockianus*	*T. tengchongensis*	*T. aquaticus*
T. igniterrae	100	26.7	27.5	28.6	21.8	25.9	25.8	25.8	24.6	25.7	27.2	25.8	26	27.2	26.9	27	26.8
T. antranikianii	26.7	100	27.2	25.1	20.5	23.3	23.3	23.3	22.7	23.2	23.9	58.9	23.4	32.1	24.6	35.6	23.9
T. caliditerrae	27.5	27.2	100	27.1	21.2	24.5	24.5	24.5	23.6	24.4	24.8	27.5	24.5	30.1	25.4	41.2	25.1
T. sp. CCB_US3_UF1	28.6	25.1	27.1	100	21.6	25.3	25.1	25.1	24.4	25.2	25.3	25.3	25.2	26.8	26.5	26.4	25.9
T. filiformis	21.8	20.5	21.2	21.6	100	22.6	22.7	21.6	21.9	22.4	22.7	20.6	22.4	21.3	21.4	21	22.1
T. thermophilus HB8	25.9	23.3	24.5	25.3	22.6	100	89	27.8	24.4	71.7	61.5	23.4	69.9	24.6	25.3	24.5	26.4
T. thermophilus HB27	25.8	23.3	24.5	25.1	22.7	89	100	27.7	24.4	72.6	61	23.5	68.9	24.8	25.4	24.3	26.5
T. islandicus	25.8	23.3	24.5	25.1	21.6	27.8	27.7	100	24.1	27.3	27	23.7	27	24.4	25.1	24	28.2
T. oshimai	24.6	22.7	23.6	24.4	21.9	24.4	24.4	24.1	100	24.7	24.5	23.2	24.3	23.9	24	23.5	25.6
T. thermophilus JL-18	25.7	23.2	24.4	25.2	22.4	71.7	72.6	27.3	24.7	100	63.2	23.4	70.8	24.6	25.2	24.7	26.5
T. parvatiensis	27.2	23.9	24.8	25.3	22.7	61.5	61	27	24.5	63.2	100	24.5	64.6	25	26.1	24.8	26.3
T. scotoductus	25.8	58.9	27.5	25.3	20.6	23.4	23.5	23.7	23.2	23.4	24.5	100	23.7	32.6	24.6	36.3	24.3
T. thermophilus SG0.5JP17-16	26	23.4	24.5	25.2	22.4	69.9	68.9	27	24.3	70.8	64.6	23.7	100	24.9	25.5	24.8	25.9
T. amyloliquefaciens	27.2	32.1	30.1	26.8	21.3	24.6	24.8	24.4	23.9	24.6	25	32.6	24.9	100	25.5	34.4	25.5
T. brockianus	26.9	24.6	25.4	26.5	21.4	25.3	25.4	25.1	24	25.2	26.1	24.6	25.5	25.5	100	25.3	25.5
T. tengchongensis	27	35.6	41.2	26.4	21	24.5	24.3	24	23.5	24.7	24.8	36.3	24.8	34.4	25.3	100	24.9
T. aquaticus	26.8	23.9	25.1	25.9	22.1	26.4	26.5	28.2	25.6	26.5	26.3	24.3	25.9	25.5	25.5	24.9	100
