# Supplementary material for: Complete Genome Analysis of Thermus parvatiensis and Comparative Genomics of Thermus spp. Provide Insights into Genetic Variability and Evolution of Natural Competence as Strategic Survival Attributes
Source: Front Microbiol. 2017 Jul 27;8:1410. doi: 10.3389/fmicb.2017.01410 (PMC5529391; doi:10.3389/fmicb.2017.01410)
Supplement: Supplementary file 4 [file Table4.PDF]

Supplementary table 4: Mobile elements harbored across *Thermus* genomes.

	Genomic Islands	IS elements		CRISPR loci	Phages
		Complete	Partial		
T. parvatiensis	3(21,338 bp; 1.06%)	5	20	1	2 intact; 1 questionable
T. thermophilus HB27	10 (122,435 bp; 5.75%)	8	31	9	0
T. thermophilus HB8	5 (98,309 bp; 4.47%)	18	22	9	0
T. thermophilus JL-18	4 (49,113 bp; 2.12%)	15	21	6	0
T. thermophilus SG0.5JP17-16	7 (69,837 bp; 3.03%)	1	27	6	0
T. scotoductus	9 (137,873 bp; 5.85%)	0	4	3	0
T. oshimai	7 (78,981 bp; 3.29%)	1	1	5	0
T. sp. CCB_US3_UF1	9 (126,041 bp; 5.57%)	13	0	7	0
T. aquaticus	5 (107,225 bp; 4.58%)	12	14	7	1 questionable; 2 incomplete
T. brockianus	4 (82,411 bp; 3.45%)	2	0	8	1 questionable; 1 incomplete
T. antranikianii	7 (54,028 bp; 2.50%)	0	13	0	0
T. filiformis	7 (67,339 bp; 2.82%)	8	6	9	0
T. islandicus	0	2	7	0	0
T. igniterrae	3 (46,642 bp; 2.09%)	1	10	9	0
T. caliditerrae	3 (31,833 bp; 1.44%)	4	2	4	0
T. amyloliquefaciens	6 (91,528 bp; 4.24%)	2	0	5	1 incomplete
T. tengchongensis	6 (79,217 bp; 3.09%)	5	4	4	1 incomplete
