# Supplementary material for: Complete Genome Analysis of Thermus parvatiensis and Comparative Genomics of Thermus spp. Provide Insights into Genetic Variability and Evolution of Natural Competence as Strategic Survival Attributes
Source: Front Microbiol. 2017 Jul 27;8:1410. doi: 10.3389/fmicb.2017.01410 (PMC5529391; doi:10.3389/fmicb.2017.01410)
Supplement: Supplementary file 5 [file Table5.PDF]

Supplementary table 5: Annotation of genes identified on the genomic island of *T. parvatiensis* plasmid pTP143 and BLASTp results with *Thermus* chromosomes (Identity ≥ 70%; e-value ≤ 1.00E-15)

Locus ID of plasmid pTP143 genes	Start coordinates	Stop coordinates	Strand	Gene annotated	Identified on chromosome of:	Locus tag	Identity	Query cover	e-value
AV541_10325	27480	27106	-	Transposase	*T. parvatiensis*	AV541_03920	98%	100%	4.00E-84
AV541_10325	27795	27499	-	hypothetical protein	*T. parvatiensis*	AV541_05845	99%	100%	2.00E-66
					T. parvatiensis	AV541_03920	99%	100%	3.00E-66
					T. parvatiensis	AV541_09480	97%	100%	5.00E-65
					T. parvatiensis	AV541_03195	99%	83%	4.00E-54
AV541_10330	28135	29259	+	hypothetical protein	*T. aquaticus*	TO73_0669	99%	96%	0
					T. aquaticus	TO73_1089	99%	99%	0
					T. brockianus	A0O31_01364	99%	95%	0
					T. brockianus	A0O31_01450	99%	95%	0
					T. brockianus	A0O31_00292	99%	95%	0
					T. parvatiensis	AV541_09845	100%	100%	0
					T. parvatiensis	AV541_00530	100%	100%	0
					T. parvatiensis	AV541_09985	99%	100%	0
					T. parvatiensis	AV541_07495	99%	100%	0
					T. parvatiensis	AV541_02220	99%	100%	0
					T. parvatiensis	AV541_00535	99%	100%	0
					T. parvatiensis	AV541_08460	99%	100%	0
					T. parvatiensis	AV541_06085	99%	100%	0
					T. parvatiensis	AV541_06030	99%	100%	0
					T. parvatiensis	AV541_02275	99%	100%	0
					T. parvatiensis	AV541_01800	99%	100%	0
					T. parvatiensis	AV541_04020, AV541_04025	99%	100%	0
					T. scotoductus	TSC_c16690	99%	95%	0
					T. scotoductus	TSC_c04220	99%	95%	0
					T. scotoductus	TSC_c18490	99%	95%	0
					T. scotoductus	TSC_c04670	99%	100%	0
AV541_10335	30342	29314	-	hypothetical protein					
AV541_10365, AV541_10370	33892	34782	+	putative ribonucleoprotein-related protein					
AV541_10375	34810	35400	+	hypothetical protein					
AV541_10380	35422	35847	+	hypothetical protein					
AV541_10385, AV541_10390	35946	37124	+	Exonuclease SbcD					
AV541_10385, AV541_10390	37105	39822	+	Exonuclease SbcC					
AV541_10395	40108	39830	-	hypothetical protein					
AV541_10400	41163	40507	-	hypothetical protein	*T. scotoductus*	TSC_c12620	99%	78%	6.00E-156
AV541_10405	41528	41830	+	FIG00789891: hypothetical protein					
AV541_10410	41956	42486	+	ATPase component BioM of energizing module of biotin ECF transporter	*T. scotoductus*	TSC_c12600	90%	98%	5.00E-107
AV541_10410	42456	43184	+	ATPase	*T. scotoductus*	TSC_c12600	99%	99%	2.00E-171
AV541_10410	43166	43300	+	hypothetical protein					
AV541_10460, AV541_10465	52497	51226	-	Mobile element protein	HB8	TTHA1018	96%	99%	0
					HB8	TTHA0234	96%	99%	0
					HB8	TTHA1270	96%	96%	0
					SG0.5JP17-16	Ththe16_1501	93%	90%	0
					JL-18	TtJL18_1114	99%	96%	0
					JL-18	TtJL18_1111	98%	93%	0
					JL-18	TtJL18_0252	99%	96%	0
					JL-18	TtJL18_0290	99%	96%	0
					CCB_US3_UF1	TCCBUS3UF1_17350	97%	96%	0
					CCB_US3_UF1	TCCBUS3UF1_17340	97%	96%	0
					CCB_US3_UF1	TCCBUS3UF1_17300	97%	96%	0
					CCB_US3_UF1	TCCBUS3UF1_17470	97%	96%	0
					CCB_US3_UF1	TCCBUS3UF1_17530	97%	87%	0
					CCB_US3_UF1	TCCBUS3UF1_18780	97%	96%	0
					CCB_US3_UF1	TCCBUS3UF1_2490, TCCBUS3UF1_2500	97%	96%	0
					CCB_US3_UF1	TCCBUS3UF1_2470	97%	96%	0
					CCB_US3_UF1	TCCBUS3UF1_11500	97%	96%	0
					CCB_US3_UF1	TCCBUS3UF1_2070	97%	96%	0
					CCB_US3_UF1	TCCBUS3UF1_1280	97%	96%	0
					CCB_US3_UF1	TCCBUS3UF1_730	97%	96%	0
					T. parvatiensis	AV541_04485	97%	94%	0
					T. scotoductus	TSC_c12380	73%	98%	4.00E-105
					T. scotoductus	TSC_c15890	73%	99%	0
AV541_10470	52752	53783	+	Mobile element protein	*T. aquaticus*	TO73_1128	87%	100%	0
					HB27	TT_C1169	89%	100%	0
AV541_10530	67385	68416	+	Mobile element protein	T. aquaticus	TO73_1128	87%	100%	0
					HB27	TT_C1169	89%	100%	0
