# Supplementary material for: Complete Genome Analysis of Thermus parvatiensis and Comparative Genomics of Thermus spp. Provide Insights into Genetic Variability and Evolution of Natural Competence as Strategic Survival Attributes
Source: Front Microbiol. 2017 Jul 27;8:1410. doi: 10.3389/fmicb.2017.01410 (PMC5529391; doi:10.3389/fmicb.2017.01410)
Supplement: Supplementary file 6 [file Table6.PDF]

Supplementary table 6: Genes and pathways annotated on metagenomic islands mapped on chromosome and plasmid of *T. parvatiensis*.

Metagenomic Island		Coordinates	Genes Annotated	Pathway	Copy number
T. parvatiensis chromosome	MGI 1	59911 - 66456	N-acetylglutamate synthase	Arginine biosynthesis	1
			Arginosuccinate lyase	Arginine biosynthesis	1
			Arginosuccinate synthase	Arginine biosynthesis	2
			Hpothetical protein		2
T. parvatiensis chromosome	MGI 2	366183-387139	Glycerol-3-phosphate dehydrogenase [NAD(P)+]	Glycerolipid and Glycerophospholipid metabolism	1
			Hypothetical protein		6
			Beta-Keto adipate enol-lactone hydrolase	Protocatechuate branch of beta-ketoadipate pathway	1
			Putative iron-sulfur cluster assembly scaffold protein	Iron-sulfur cluster assembly	1
			Cysteine desulfurase	Iron-sulfur cluster assembly	1
			Lead, cadmium, zinc and mercury transporting ATPase	Copper transport system	1
			Multicopper oxidase	Copper homeostasis	1
			Putative membrane protein		1
			Methyl transfersae type II		1
			S-layer repressor		1
			Putative periplasmic or exported protein		1
T. parvatiensis chromosome	MGI 3	1017478-1048913	Putative two-component system sensor kinase		2
			Puative two-component response regulator		1
			Putative membrane protein		1
			Hypothetical protein		6
			ATP dependent DNA helicase Uvr D/Pcr A	DNA repair	2
			Polyphosphate kinase	Purine conversions; High affinity phosphate transporter and control of PHO regulon; Phosphate metabolism	1
			Succinate semialdehyde dehydrogenase		1
			Two-component response regulator		1
			AttH protein		1
			Fructokinase	Fructose utilization; Sucrose utilization; Mannitol utilization	1
			HD hydrolase domain		1
			Signal-transducing histidine kinase homolog		1
			Sensory transduction histidine kinase		1
			Transcriptional regulator, MarR family		1
			Multidrug resistance protein B		1
			Allophanate hydrolase 2 subunit 1		1
			3-Ketoacyl-CoA thiolase	Serine-glyoxylate cycle; Phenylalkanoid acid degradation	1
			Thymidylate kinase		2
			Quinolinate phosphoribosyltransferase		2
			Quinolinate synthetase		1
			L-aspartate oxidase		1
			Bacterioferritin		1
			Transcriptional regulator, HxDR family		1
			Fructose 1,6-bisphosphatase, type V, archaeal		1

Metagenomic Island		Coordinates	Genes Annotated	Pathway	Copy number
T. parvatiensis chromosome	MGI 4	1602597-1626862	Short chain dehydrogenase/reductase	Transcription repair cluster	1
			Hypothetical protein		4
			Probable methylmalonyl-CoA epimerase		1
			Fumarate/succinate/L-aspartate dehydrogenases		1
			Ferredoxin		1
			Chorismate mutase I	Chorismate synthesis	1
			Twin-arginine translocation protein Tat C	Twin-arginine translocation system	1
			Twin-arginine translocation protein Tat A	Twin-arginine translocation system	1
			N-acetylglucosamine-1-phosphate uridyltransferase	Transcription repair cluster; Sialic acid metabolism	1
			Prolipoprotein diacylglycerol transferase	Lipoprotein biosynthesis	1
			Probable membrane protein NMA1128		1
			Tungsten containing aldehyde ferredoxin oxidoreductase		1
			Glycerol 3-phosphate ABC transporter		1
			N-acetyl D-glucosamine ABC transport system		1
			Sugar ABC transporter, permease protein		1
			Glycerol 3-phosphate ABC transporter		2
			Tsa B (Bacterial RNA metabolizing Zn dependent hydrolases)	Bacterial RNA metabolizing Zn dependent hydrolases	1
			3-polyprenyl-4-hydroxybenzoate carboxy lyase		1
			Endonuclease		1
			Serine/threonine protein kinase		1
			Thioredoxin reductase	Pyrimidine conversions	1

T. parvatiensis chromosome	MGI 5	1713444-1743224	Probable serine protease		1
			UDP-N-acetyl muramoyl alanyl-D-glutamate-L-ornithine ligase	Peptidoglycan biosynthesis	1
			Transcriptional regulator, TetR family		1
			Hypothetical protein		17
			Putative membrane protein		1
			2-nitropropane dioxygenase		1
			Major facilitator superfamily MFS-1		1
			Mannose-6-phosphate 3 isomerase	Mannose metabolism	1
			Ribulose-phosphate 3 epimerase	Conserved gene cluster associated with Met-tRNA foromyltransferase	1
			Transketolase	Calvin-Benson cycle; Pentose phosphate pathway	1
			Integrase		1
			Type III restriction-modification system	Restriction modification system	1
			DNA binding protein		1
			DNA primase, phage associated		1
			DEAD-box ATP-dependent RNA helicase csh A		1
			Molybdopterin biosynthesis protein Moc A	Molybdenum cofactor biosynthesis	2
			Permease		1
			Endonuclease III	Base excision repair; Control of cell elongation	1

Metagenomic Island		Coordinates	Genes Annotated	Pathway	Copy number
Plasmid pTP143	MGI 1	17130-25357	DNA reverse gyrase		2
			Hypothetical protein		3
Plasmid pTP143	MGI 2	38267-42655	Exonuclease sbc D		1
			Exonuclease sbc C		1
			Hypothetical protein		3
			ATPase component BioM of energizing module of biotin ECF transporter		1
Plasmid pTP143	MGI 3	110369-113154	Hypothetical protein		1
			Plasmid stability protein Stb B		1
			Phytoene desaturase, neurosporene or lycopene producing		2
			Isopentenyl-diphosphate delta isomerase		3
Plasmid pTP143	MGI 4	117415-124334	Chromosome (plasmid) partitioning protein (Par B)		1
			Soj protein		1
			Hypothetical protein		5
			Assimilatory nitrate reductase large subunit		1
			Hypothetical membrane spanning protein		1
Plasmid pTP143	MGI 5	129101-133911	Radical SAM domain heme biosynthesis protein		1
			Cytochrome C-552 cytochrome c nitratereductase precursor		1
			Transcriptional regulator, MarR family		1
			Peptide methionine sulfoxide reductase Msr A		1
			Probable uro porphyrin III C-methyltransferase		1
			Hypothetical protein		3
			NADh dehydrogenase		1
