# Supplementary material for: Complete Genome Analysis of Thermus parvatiensis and Comparative Genomics of Thermus spp. Provide Insights into Genetic Variability and Evolution of Natural Competence as Strategic Survival Attributes
Source: Front Microbiol. 2017 Jul 27;8:1410. doi: 10.3389/fmicb.2017.01410 (PMC5529391; doi:10.3389/fmicb.2017.01410)
Supplement: Supplementary file 7 [file Table7.PDF]

Supplementary table 7: Predicted structure motifs, sequence families and superclass for CRISPR repeats on the basis of identity with known consensus repeats (based on 40 sequence families and 33 structure motifs).

Species	Structure motifs	Sequence families	Superclass
T. parvatiensis	motif 9	family 37	D
T. thermophilus HB27	motif 5, motif 9, motif 25	family 31, family 37	D, E
T. thermophilus HB8	motif 2, motif 5, motif 25, motif 31	family 4, family 31	C, D, E
T. thermophilus JL-18	motif 1, motif 2, motif 23	family 4, family 18	B, C, E
T. thermophilus SG0.5JP17-16	motif 9, motif 24	family 37	C, D, E
T. scotoductus	motif 1, motif 2	family 18, family 21	B, C
T. oshimai	motif 25	family1, family 18, family 21	A, B, C, D
T. sp. CCB_US3_UF1	motif 5, motif 25	family 1, family 21, family 31	A, C, D, E
T. aquaticus	motif 25, motif 18	family 1, family 18	A, B
T. brockianus	motif 2, motif 5	family1, family 4, family 21, family31	C, D, E
T. antranikianii	-	-	-
T. filiformis	motif 2, motif 20, motif 25	family 1, family 4, family 21	A, B, C, D
T. islandicus	-	-	-
T. igniterrae	motif 5, motif 25	family 18, family 2, family 31	B, C, D
T. caliditerrae	motif 5	family 31	E
T. amyloliquefaciens	motif 18, motif 25	family1, family 18	A, B
T. tengchongensis	motif 6, motif 24	family 1	A, B, E
