# Supplementary material for: Complete Genome Analysis of Thermus parvatiensis and Comparative Genomics of Thermus spp. Provide Insights into Genetic Variability and Evolution of Natural Competence as Strategic Survival Attributes
Source: Front Microbiol. 2017 Jul 27;8:1410. doi: 10.3389/fmicb.2017.01410 (PMC5529391; doi:10.3389/fmicb.2017.01410)
Supplement: Supplementary file 8 [file Table8.PDF]

Supplementary table 8: Number of *Thermus* genomes classified into subsequent CRISPR superclasses, sequence families and structure motifs on the basis of CRISPR repeat identities with known CRISPR repeat sequences.

Superclass	No of species	Family	No of species	Motif	No of species
Superclass A	6	Family1	7	Motif 1	2
Superclass B	8	Family 2	1	Motif 2	5
Superclass C	9	Family 4	4	Motif 5	6
Superclass D	9	Family 18:	6	Motif 6	1
Superclass E	8	Family 21	5	Motif 9	3
		Family 31:	6	Motif 18	2
		Family 37	3	Motif 20	1
				Motif 23	1
				Motif 24	2
				Motif 25	8
				Motif 31	1
