# Supplementary material for: Complete Genome Analysis of Thermus parvatiensis and Comparative Genomics of Thermus spp. Provide Insights into Genetic Variability and Evolution of Natural Competence as Strategic Survival Attributes
Source: Front Microbiol. 2017 Jul 27;8:1410. doi: 10.3389/fmicb.2017.01410 (PMC5529391; doi:10.3389/fmicb.2017.01410)
Supplement: Supplementary file 9 [file Table9.PDF]

Supplementary table 9: BLAST (identity >20 nt, e-value <1) hits of CRISPR spacers to viral sequence database; NI: not identified.

Organism	CRISPR Locus	Spacer	Phage	Family	Identity	E-value	Score
T. filiformis	2	3	Thermus phage P23-77	*Sphaerolipoviridae*	31/31	1e-08	61.9
	2	23	Thermus phage P23-77	*Sphaerolipoviridae*	32/32	3e-09	63.9
	3	2	Simian adenovirus sp	*Adenoviridae*	24/25	0.021	42.1
	3	5	Salmonella phage 40	*Myoviridae*	21/21	0.017	42.1
	3	7	Ralstonia phage RSB2 DNA	*Podoviridae*	20/20	0.072	40.1
	3	4	HIV-1 isolate 04MZCMM28 from Mozambique reverse transcriptase (pol) gene	*Herpesviridae*	20/20	0.065	40.1
T. igniterrae	2	8	Dickeya sp phage	*Myoviridae*	20/20	0.055	40.1
	2	13	Arthrobacter sp phage	*Myoviridae*	21/22	0.80	36.2
	2	21	Xanthomonas oryzae phage OP2	*Myoviridae*	21/22	0.75	36.2
	3	1	Pseudomonas sp phage	*Cystoviridae*	21/22	0.91	36.2
	6	1	Streptomyces phage mu1/6	*Siphoviridae*	22/23	0.27	38.2
	7	5	Arthrobacter sp phage	*Myoviridae*	21/22	0.080	36.2
	7	12	Xanthomonas oryzae phage OP2 DNA	*Myoviridae*	21/22	0.75	36.2
	8	1	Pandoravirus inopinatum isolate KlaHel	*Pandoraviridae*	23/23	8e-04	46.1
	8	12	Micropterus salmoides reovirus isolate 1001 segment 4	*Reoviridae*	20/20	0.051	40.1
	8	22	Thermus thermophilus phage IN93	*Sphaerolipoviridae*	28/29	5e-05	50.1
T. oshimai	3	42	Thermus phage P23-77	*Sphaerolipoviridae*	22/23	0.19	38.2
	3	43	Thermus thermophilus phage IN93	*Sphaerolipoviridae*	31/32	8e-07	56.0
	3	8	Bear Canyon virus strain AV B0300052 nucleocapsid protein gene	*Arenaviridae*	21/22	0.75	36.2
	3	25	Thermus thermophilus phage IN93	*Sphaerolipoviridae*	32/32	3e-09	63.9
	3	27	Aeropyrum pernix K1		22/23	0.19	38.2
T. scotoductus	2	7	Thermus thermophilus phage IN93	*Sphaerolipoviridae*	32/32	3e-09	63.9
	2	8	Thermus thermophilus phage IN93	*Sphaerolipoviridae*	32/32	3e-09	63.9
	2	16	Emiliania huxleyi virus	*Phycodnaviridae*	22/23	0.19	38.2
	2	30	Thermus thermophilus phage IN93	*Sphaerolipoviridae*	21/21	0.011	42.1
	2	31	Thermus thermophilus phage IN93	*Sphaerolipoviridae*	61/61	1e-08	61.9
T. sp. CCB_US3_UF1	2	13	Rhodobacter sp phage	*Siphoviridae*	22/22	0.005	44.1
T. tengchongensis	4	32	Pandoravirus dulcis	*Pandoraviridae*	20/20	0.065	40.1
	4	33	Connecticut virus nucleoprotein	NI	29/31	0.001	46.1
	4	44	Aotine herpesvirus 1 strain S34E	*Herpesviridae*	23/23	0.001	46.1
	5	3	Aeromonas phage Ahp1	*Podoviridae*	22/23	0.27	38.2
	5	8	Actinoplanes phage phiAsp2	*Siphoviridae*	23/24	0.072	40.1
	5	10	Actinoplanes phage phiAsp2	*Siphoviridae*	23/24	0.072	40.1
T. thermophilus HB8 plasmid pTT27	2	5	Streptomyces phage Chymera	*Siphoviridae*	20/20	0.065	40.1
	3	2	Thermus phage OH3	*Inoviridae*	23/23	8e-04	46.1
	3	4	Thermus phage OH3	*Inoviridae*	20/20	0.048	40.1
	3	10	Epstein-Barr virus (EBV) genome	*Herpesviridae*	20/20	0.048	40.1
	3	18	Suid alphaherpesvirus 1	*Herpesviridae*	21/21	0.014	42.1
	4	8	Actinoplanes phage phiAsp2	*Siphoviridae*	21/22	0.96	36.2
	4	9	Porcine astrovirus 5 clone PFP-33 ORF1ab	*Astroviridae*	21/22	0.96	36.2

			gene				
T. thermophilus HB27 plasmid pTT27	2	5	Pseudomonas phage phiAH14b	*Cystoviridae*	20/20	0.058	40.1
	1	3	Thermus phage TMA	*Myoviridae*	28/31	0.31	38.2
	1	9	Streptomyces phage Chymera	*Siphoviridae*	20/20	0.065	40.1
	1	6	Pandoravirus dulcis	*Pandoraviridae*	24/26	0.96	36.2
	1	6	Mycobacterium phage Pipefish	*Siphoviridae*	24/26	0.96	36.2
	1	7	Thermus thermophilus phage IN93	*Sphaerolipoviridae*	27/29	0.015	42.1
	1	6	Thermus thermophilus phage IN93	*Sphaerolipoviridae*	36/36	2e-11	71.9
	3	1	Thermus thermophilus phage IN93	*Sphaerolipoviridae*	33/34	7e-08	60.0
	3	2	Pseudomonas phage PAJU2	*Siphoviridae*	21/22	0.96	36.2
	3	9	Rhodococcus phage ReqiPine5	*Siphoviridae*	22/23	0.23	38.2
T. thermophilus JL-18 plasmid pTTJL1801	1	2	Thermus thermophilus phage IN93	*Sphaerolipoviridae*	32/33	2e-07	58.0
	1	6	Thermus thermophilus phage IN93	*Sphaerolipoviridae*	31/31	1e-08	61.9
	1	22	Thermus thermophilus phage IN93	*Sphaerolipoviridae*	31/32	8e-07	56.0
	2	7	Bovine herpesvirus type 1.2	*Herpesviridae*	27/29	0.015	42.1
	2	4	Delftia phage IME-DE1	*Podoviridae*	25/27	0.23	38.2
	2	8	Human herpesvirus 2 isolate 15 genome assembly	*Herpesviridae*	20/20	0.065	40.1
	2	20	Bovine papular stomatitis virus	*Poxviridae*	21/22	0.96	36.2
	2	7	Thermus sp phage	NI	25/26	0.004	44.1
	2	19	Mycobacterium sp phage	NI	21/22	0.91	36.2
T. thermophilis SG0.5JP17-16	1	3	Tortoise ranavirus isolate 1 (882/96)	*Iridoviridae*	21/21	0.016	42.1
	2	4	Bovine herpesvirus 1 strain Cooper	*Herpesviridae*	22/23	0.26	38.2
