# Supplementary figures and images for: Complete Genome Analysis of Thermus parvatiensis and Comparative Genomics of Thermus spp. Provide Insights into Genetic Variability and Evolution of Natural Competence as Strategic Survival Attributes
Source: Front Microbiol. 2017 Jul 27;8:1410. doi: 10.3389/fmicb.2017.01410 (PMC5529391; doi:10.3389/fmicb.2017.01410)

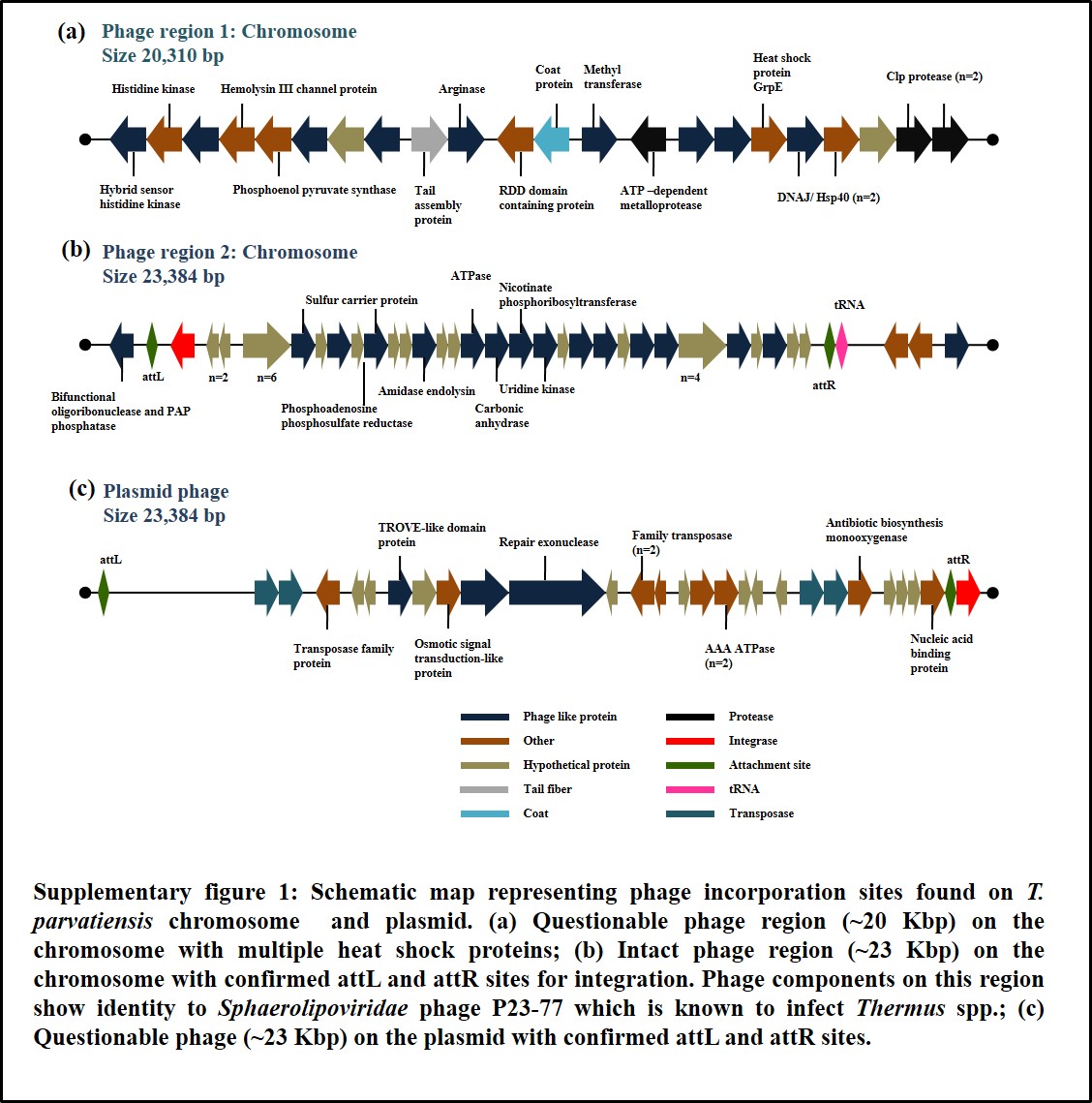

Supplement: Supplementary file 10 [file Image1.jpg]

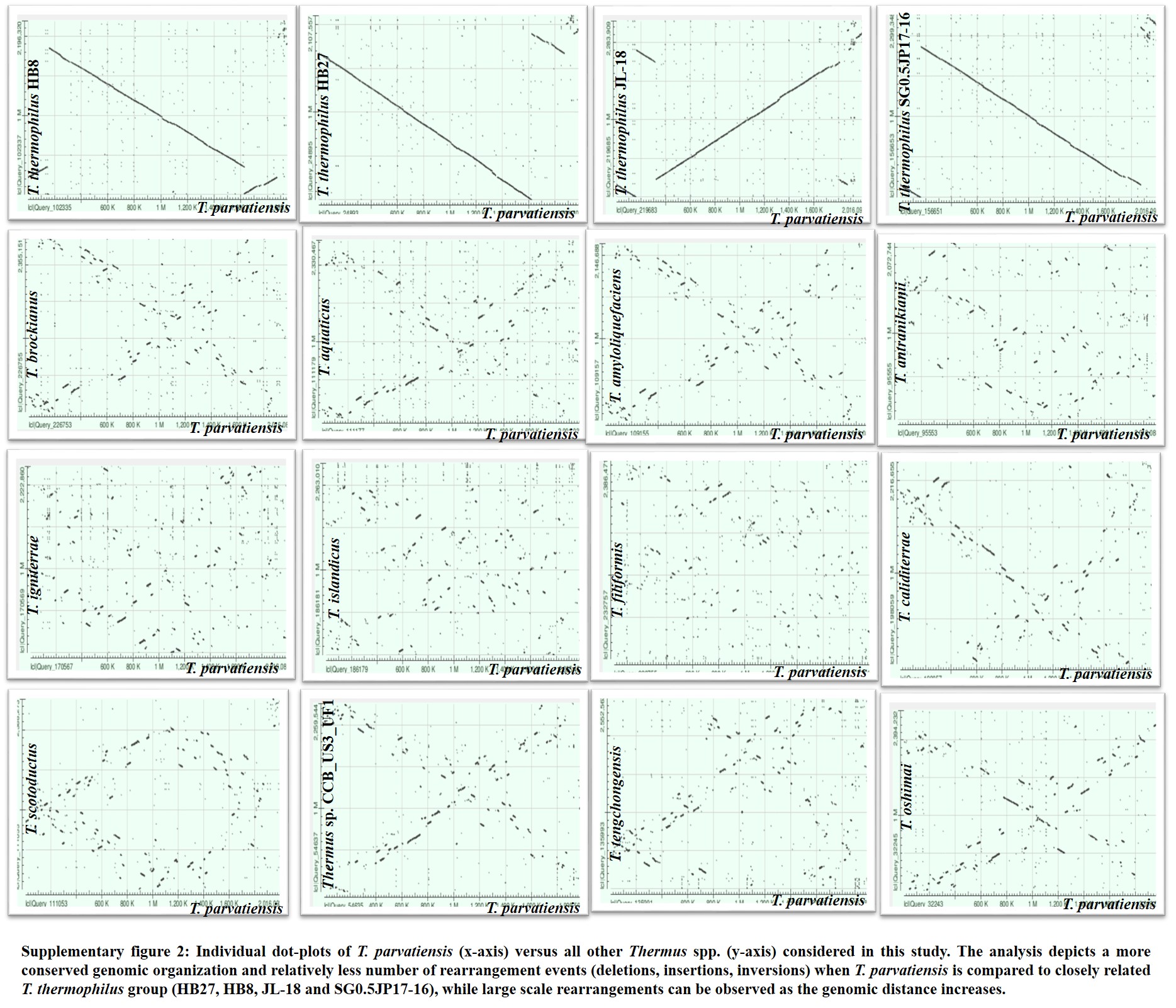

Supplement: Supplementary file 11 [file Image2.jpg]

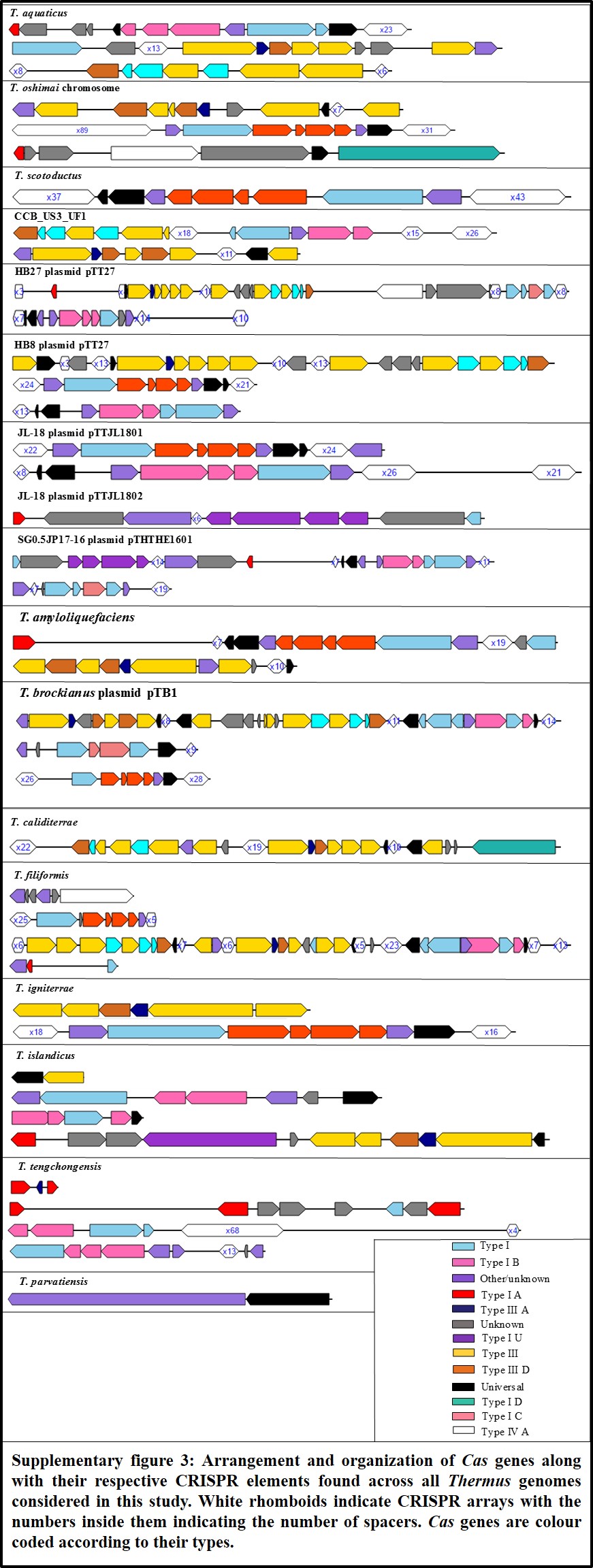

Supplement: Supplementary file 12 [file Image3.jpg]

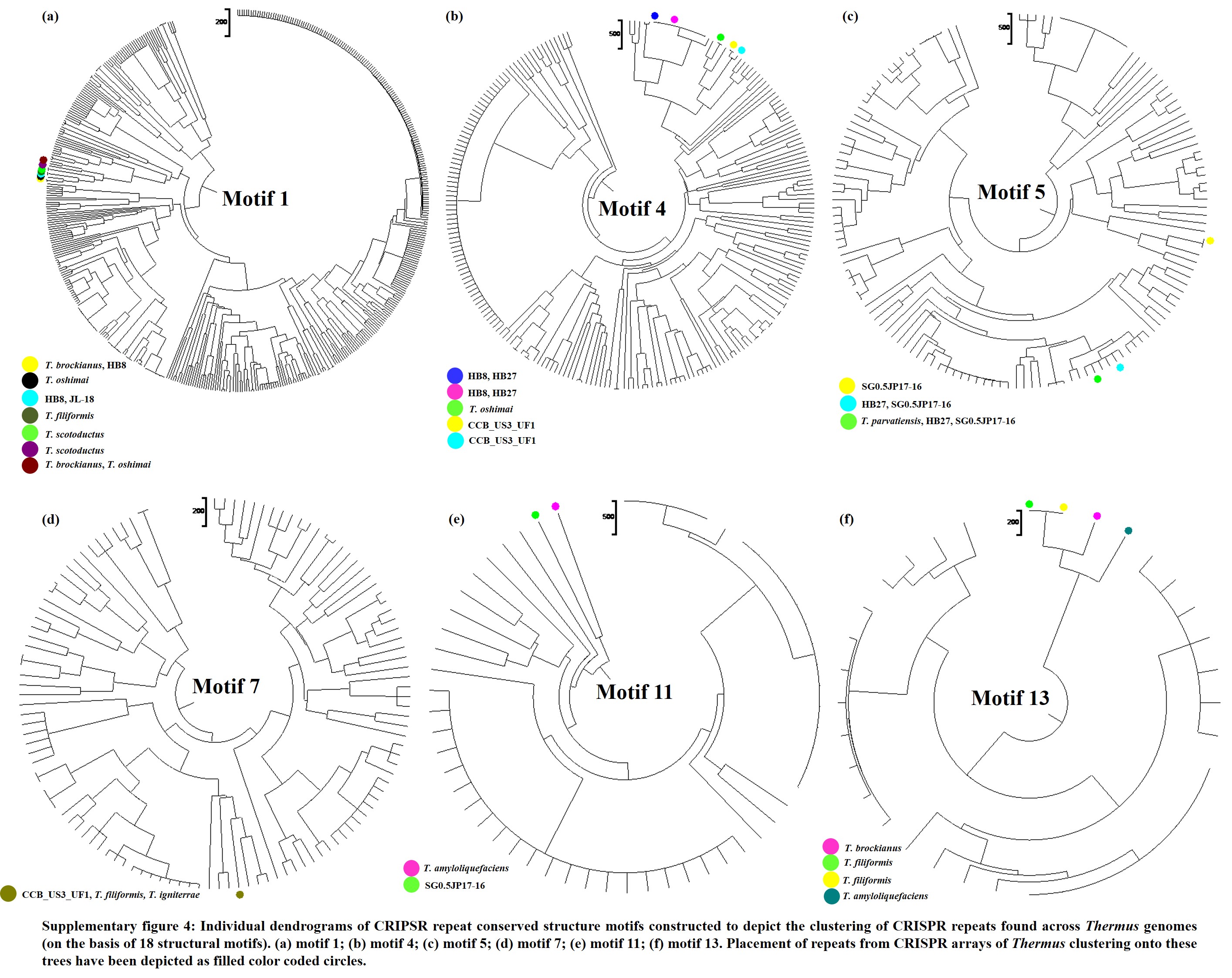

Supplement: Supplementary file 13 [file Image4.jpg]

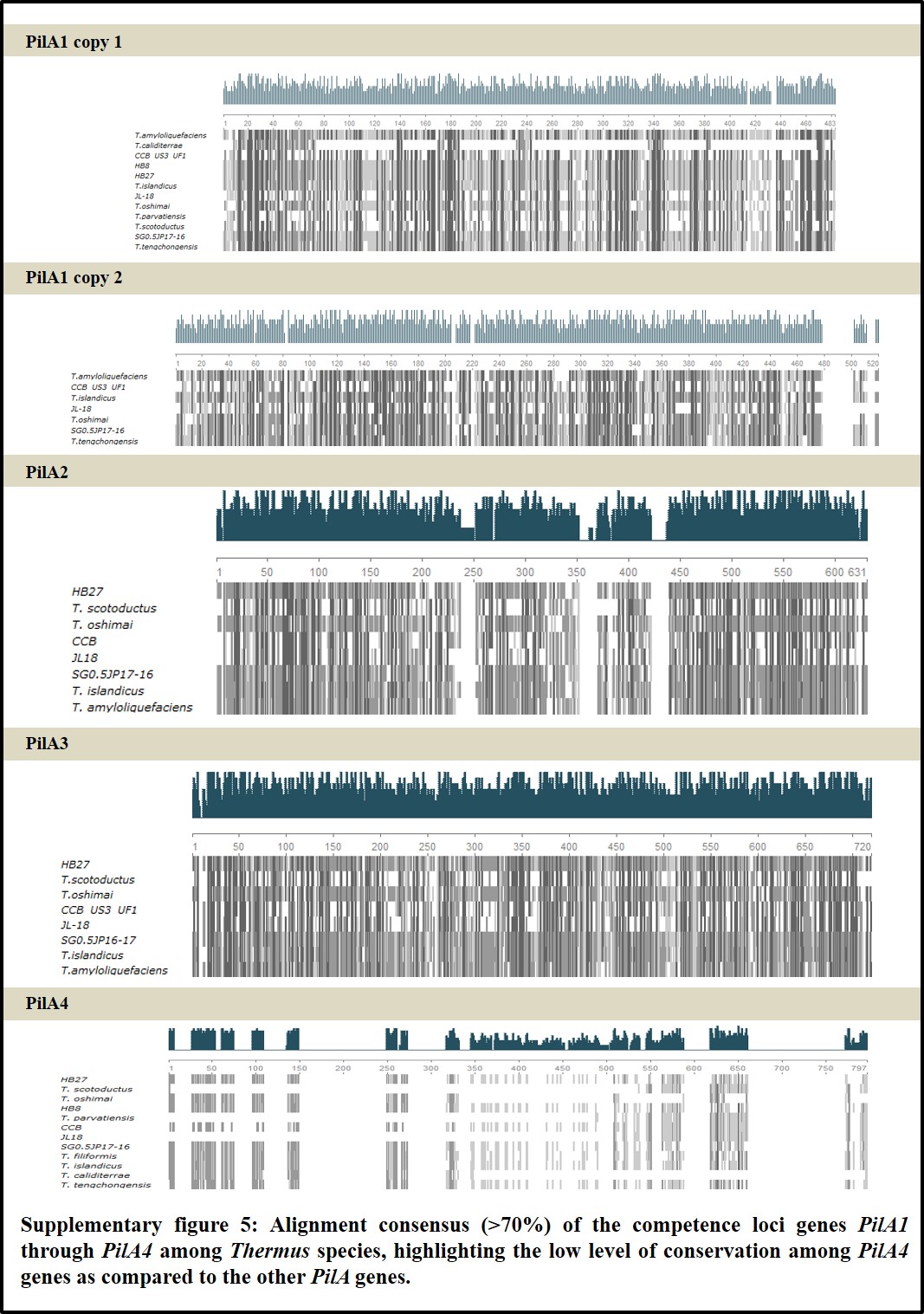

Supplement: Supplementary file 14 [file Image5.jpg]

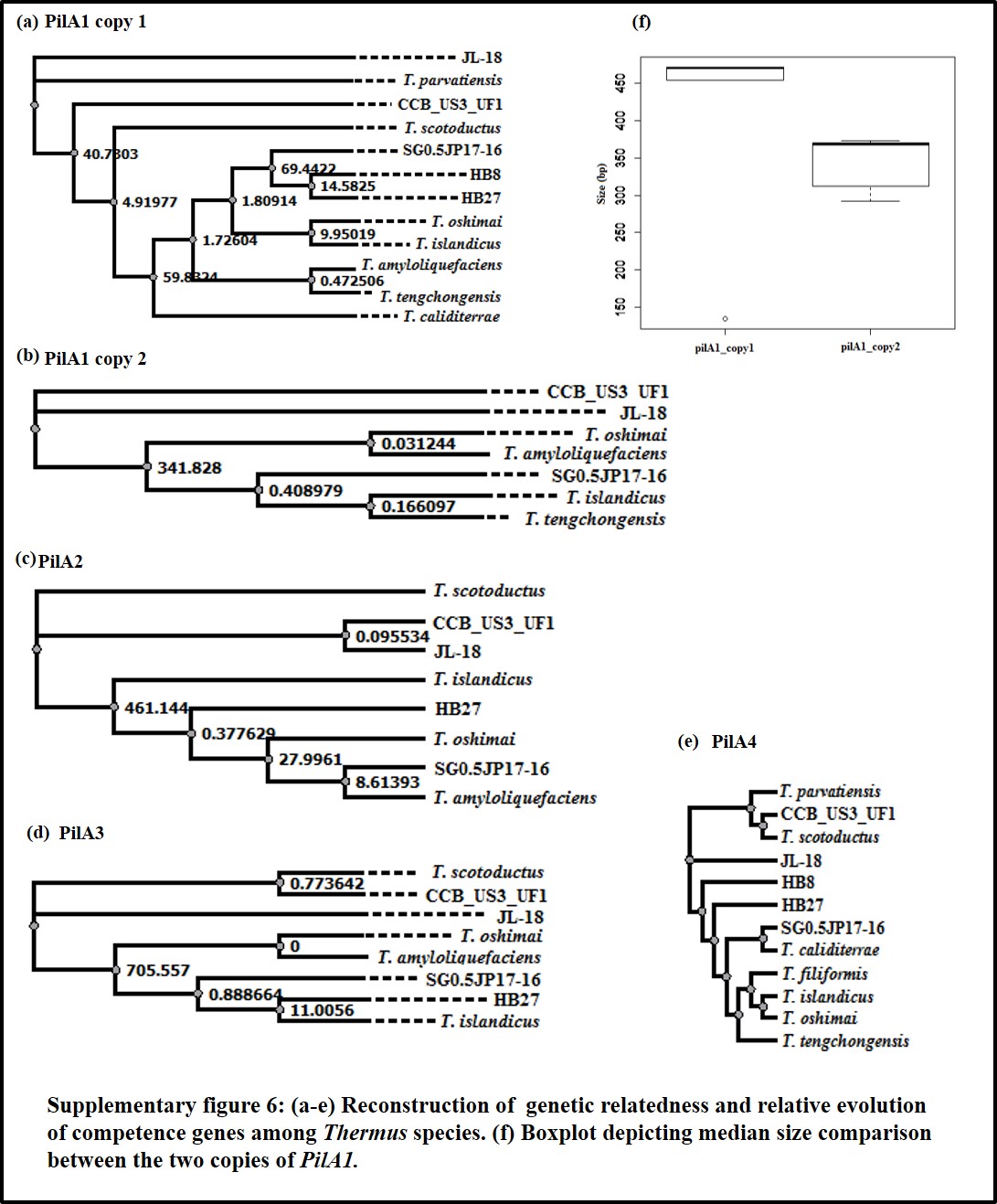

Supplement: Supplementary file 15 [file Image6.jpg]
